# Polysulfone Hollow Fiber Mixed Matrix Membrane Contactor Incorporating Fluorinated Silicon Carbide Nanoparticles for CO_2_ Absorption

**DOI:** 10.3390/membranes16100320

**Published:** 2026-09-26

**Authors:** Amin Dehghani, Masoud Rahbari-Sisakht, Amir Mansourizadeh, Ahmad Fauzi Ismail

**Affiliations:** 1 Department of Chemical Engineering, Gac. C., Islamic Azad University, Gachsaran, Iran; 2 Advanced Membrane Technology Research Centre (AMTEC), Faculty of Chemical and Energy Engineering, Universiti Teknologi Malaysia, Skudai 81310, Johor, Malaysia; 3 Department of Chemistry, Faculty of Science, Institut Teknologi Sepuluh Nopember (ITS), Kampus ITS Sukolilo, Surabaya 60111, Indonesia

**Keywords:** carbon dioxide capture, gas–liquid membrane contactor, polysulfone hollow fiber membrane, fluorinated silicon carbide, mixed-matrix membrane, hydrophobicity

## Abstract

Carbon dioxide (CO_2_) emissions are a major driver of global climate change, creating an urgent need for efficient carbon capture technologies. Gas–liquid membrane contactors (GLMCs) offer high interfacial area and operational flexibility, but membrane pore wetting can limit their long-term performance. In this study, fluorinated silicon carbide (F-SiC) nanoparticles were incorporated into polysulfone (PSf) hollow fiber membranes to improve hydrophobicity, wetting resistance, and CO_2_ absorption performance. Mixed-matrix membranes containing 0, 2, 4, and 6 wt.% F-SiC were fabricated by wet phase inversion. CO_2_ absorption was evaluated using distilled water and 1 M NaOH as absorbents. F-SiC incorporation increased the water contact angle from 77.5° to 107.0° and the critical entry pressure of water (CEPw) from 4.0 to 10.0 bar. The membrane containing 4 wt.% F-SiC (S4) showed the highest CO_2_ absorption fluxes of 7.6 × 10^−4^ and 1.59 × 10^−3^ mol m^−2^ s^−1^ using distilled water and 1 M NaOH, respectively. Long-term stability tests showed that the onset of flux decline was delayed from 35 h for the pristine membrane to 125 h for S4, while the flux loss after 145 h was reduced from 44.44% to 12.25%, demonstrating excellent resistance to pore wetting. These results highlight the potential of F-SiC-modified PSf hollow fiber membranes for efficient and durable CO_2_ capture in GLMC applications.

## 1. Introduction

The continuous rise in CO_2_ levels in the atmosphere is a major contributor to global warming and climate change [1]. Effective capture and separation of CO_2_ from industrial flue gases and other emissions is therefore crucial [2]. Among the available technologies, GLMCs have gained considerable attention due to their high surface-area-to-volume ratio, modular design, and ability to decouple gas and liquid flow rates [3]. These systems enable efficient mass transfer without the limitations of traditional packed columns, such as flooding, channeling, or pressure drop, making them suitable for compact and high-performance CO_2_ capture units.

Hollow fiber membranes are the most widely employed configurations in GLMCs because of their high packing density, mechanical flexibility, and ease of fabrication. PSf, in particular, is a commonly used polymer due to its excellent thermal stability, chemical resistance, and film-forming properties [4]. However, conventional PSf membranes exhibit limited surface hydrophobicity, making them prone to pore wetting during operation with aqueous absorbents [5,6]. Pore wetting significantly reduces the effective gas–liquid contact area, leading to decreased CO_2_ absorption efficiency and long-term operational instability [7]. Improving the hydrophobicity, surface roughness, and overall pore structure of PSf membranes is therefore essential for enhancing their performance in GLMCs.

Beyond conventional post-combustion flue gas treatment, gas–liquid membrane contactors are increasingly being explored for more advanced and demanding separation applications. One emerging direction is direct air capture (DAC) of CO_2_, where membrane-based air–liquid contactors offer a compact, modular alternative to conventional packed-tower DAC systems, owing to their high interfacial area per unit volume and their compatibility with a wide range of aqueous capture solvents [8]. Related contactor-based configurations have also been extended to CO_2_ mineralization processes, in which membrane contactors are used to precipitate solid carbonate products for permanent carbon storage in energy-intensive industries [9]. In parallel, membrane contactors employing aqueous-amine solvents have attracted growing interest for CO_2_-rich natural gas sweetening and biogas upgrading, applications that demand membranes with both high wetting resistance and long-term chemical stability under continuous solvent exposure [10]. These emerging applications place increasingly stringent demands on membrane wetting resistance and mechanical durability—the same structure–property attributes targeted in the present work—underscoring the broader relevance of fluorinated, filler-modified hollow fiber membranes beyond conventional flue gas CO_2_ capture.

In parallel with contactor-scale process innovation, the broader mixed-matrix membrane field has recently expanded toward advanced two-dimensional and framework-based nanofillers including MXenes [11] and covalent organic frameworks (COFs) [12,13] which offer highly tunable interlayer nanochannels and tailorable surface chemistry for CO_2_-selective transport. While these fillers have so far been explored primarily in dense-film gas-separation membranes rather than porous hollow fiber contactors, they represent a promising future direction for further enhancing the gas transport and anti-wetting properties of PSf-based hollow fiber membrane contactors of the type developed in this study.

Mixed-matrix membranes (MMMs), where functional inorganic nanoparticles are incorporated into a polymer matrix, offer a promising approach to tailor membrane properties for gas–liquid applications [14]. Nanoparticle additives can increase surface hydrophobicity, enhance mechanical strength, modify pore architecture, and improve chemical and thermal stability. Rezaei et al. [15] fabricated porous asymmetric PVDF/montmorillonite (MMT) hollow fiber MMMs with different MMT nanoparticle loadings using the wet phase inversion method for CO_2_ absorption in gas–liquid membrane contactors. The incorporation of MMT increased surface roughness and porosity while slightly reducing the mean pore size (from 34 to 22 nm at 5 wt.% MMT). In addition, the modified membranes exhibited enhanced hydrophobicity, higher liquid entry pressure (LEPw), and improved mechanical strength, leading to superior resistance against membrane wetting. Performance evaluation demonstrated that CO_2_ absorption flux increased with MMT loading, reaching 1.89 × 10^−3^ mol m^−2^ s^−1^ at 5 wt.% MMT, which was 48.7% higher than that of the pristine PVDF membrane and exceeded the performance of a commercial PVDF membrane. Furthermore, the optimized membrane maintained a nearly constant absorption flux during a 350 h long-term test. Zhang et al. [16] developed a PVDF-based MMM contactor incorporating a hierarchical core–shell Cu@4A composite filler to enhance SO_2_ removal. The Cu@4A filler simultaneously modified the membrane structure and chemical environment by improving gas permeability, increasing SO_2_ adsorption and oxidation sites, and creating facilitated transport pathways at the polymer–filler interface. These effects significantly enhanced both physical and chemical SO_2_ absorption. Of all the fabricated membranes, the formulation containing 40 wt.% Cu@4A and 10 wt.% PVDF demonstrated the highest separation performance, achieving an SO_2_ removal efficiency of 73.6% and an absorption flux of 9.1 × 10^−4^ mol m^−2^ s^−1^ at a liquid flow rate of 30 L h^−1^.

More recently, PSf-based mixed-matrix membranes incorporating fluorinated silica have also been explored for CO_2_ absorption in gas–liquid membrane contactors, offering a closer structural comparison to the present work than the PVDF-based systems discussed above. Yousefinejad et al. [17] fabricated mixed matrix PSf hollow fiber membranes containing fluorinated SiO_2_ nanoparticles (0, 2, 4, and 6 wt%) via wet phase inversion and evaluated their CO_2_ absorption performance in a GLMC. Increasing nanoparticle loading raised dope viscosity and slowed phase inversion, producing thicker, sponge-like structures with fewer macrovoids, although nanoparticle agglomeration and partial pore blockage were observed at the highest (6 wt%) loading. Surface roughness increased substantially with loading (24.2 to 83.0 nm), while water contact angle and critical entry pressure of water rose from 79° to 102° and 4.5 to 8.5 bar, respectively, confirming enhanced hydrophobicity and wetting resistance. The 4 wt% membrane (PSf-4) exhibited the largest mean pore size and highest effective surface porosity, translating into the highest CO_2_ absorption flux and lowest mass-transfer resistance among the series, using both distilled water and 1 M NaOH as absorbents.

In a related study, Sadat et al. [18] fabricated superhydrophobic PSf hollow fiber membranes surface-coated with fluorinated SiO_2_ nanoparticles (synthesized using PFDTES) at 0.5, 1.0, and 1.5 wt% coating concentrations. Surface roughness increased from 18 to 35 nm with coating concentration, while water contact angle rose from 82° to 149° and critical entry pressure of water from 5.0 to 9.5 bar. CO_2_ absorption tests using distilled water and 1 M NaOH as physical and reactive absorbents, respectively, showed that flux increased with absorbent flow rate for all membranes due to reduced liquid-phase mass-transfer resistance. The membrane coated with 1.0 wt% fluorinated SiO_2_ (M1) performed the best overall, achieving maximum fluxes of 1.61 × 10^−3^ mol m^−2^ s^−1^ with 1 M NaOH and 7.33 × 10^−4^ mol m^−2^ s^−1^ with distilled water, and reduced total mass-transfer resistance by approximately 30% relative to the pristine membrane.

Although both studies demonstrate the effectiveness of fluorinated silica modification in PSf-based membranes for enhancing hydrophobicity, wetting resistance, and CO_2_ absorption performance, they differ from the present work either in the nature of the nanoparticle incorporation strategy (surface coating versus bulk mixed-matrix blending) or in the absence of a combined mechanical stability and long-term wetting-resistance evaluation. The present study builds on this body of work by systematically incorporating fluorinated SiC nanoparticles directly into the PSf bulk matrix and by additionally evaluating mechanical stability (collapsing pressure), long-term operational wetting resistance, and Wilson-plot-resolved mass-transfer resistance within a single, unified structure–property framework.

Among candidate nanomaterials, SiC is particularly attractive due to its high chemical inertness, thermal stability, mechanical robustness, and resistance to acidic or basic environments [19], making it suitable for long-term operation with aqueous or alkaline CO_2_ absorbents. However, pristine SiC nanoparticles are inherently hydrophilic and prone to aggregation [20], limiting their effectiveness in enhancing membrane hydrophobicity and process stability. The surface functionalization of SiC nanoparticles with fluorinated groups, such as 1H,1H,2H,2H-perfluorodecyltriethoxysilane (PFDTES), introduces low-surface-energy chains that improve nanoparticle dispersibility and impart strong hydrophobicity to the resulting membranes. The combination of fluorinated SiC nanoparticles with PSf is expected to enhance pore wetting resistance, surface roughness, and long-term chemical stability, while maintaining adequate gas permeability for CO_2_ transport.

Fluorinated silica [17,21] and fluorinated titania [22] nanoparticles have been studied as hydrophobizing fillers in gas–liquid membrane contactors. However, the chemical robustness of silica- and titania-based fillers under the alkaline conditions relevant to reactive CO_2_ absorption (e.g., NaOH or amine-based absorbents) is intrinsically limited by their core chemistry: silica is susceptible to hydrolytic dissolution and etching in strongly alkaline media, and TiO_2_ surfaces are prone to hydroxylation and photocatalytic side-reactions under prolonged aqueous exposure. SiC, by contrast, is chemically inert toward both acidic and alkaline environments [23], so fluorinating a SiC core is expected to combine an intrinsically stable inorganic phase with the wetting-resistance benefit of the fluorinated shell, rather than relying on a filler whose own core chemistry may degrade under prolonged exposure to alkaline absorbents. The use of fluorinated SiC nanoparticles in PSf-based hollow fiber membranes for GLMC CO_2_ absorption has, to our knowledge, not been previously reported, and this study evaluates both its structure–performance behavior and its functional performance under physical (distilled water) and reactive (1 M NaOH) absorption conditions.

In this study, we report the fabrication and performance evaluation of mixed-matrix polysulfone (PSf) hollow fiber membranes incorporating fluorinated silicon carbide (F-SiC) nanoparticles for CO_2_ absorption in a gas–liquid membrane contactor. The F-SiC nanoparticles were synthesized by silanization with 1H,1H,2H,2H-perfluorodecyltriethoxysilane (PFDTES) and incorporated into PSf dope solutions at different loadings (0–6 wt.%). Unlike conventional SiC-modified membranes, this study investigates the use of fluorinated SiC to simultaneously improve membrane hydrophobicity and resistance to pore wetting while maintaining effective CO_2_ mass transfer. The hollow fiber membranes were fabricated by wet phase inversion and systematically characterized in terms of morphology, surface roughness, wettability, critical entry pressure of water, pore size, porosity, gas permeability, and mechanical strength. Their CO_2_ absorption performance was then evaluated using distilled water and NaOH solutions as absorbents. The effects of F-SiC loading on membrane structure, surface properties, wetting resistance, and CO_2_ mass transfer were examined, including long-term absorption stability. This systematic analysis provides insights into the role of fluorinated SiC in controlling the structural and interfacial properties of PSf hollow fiber membranes for durable CO_2_ capture in GLMC systems.

## 2. Experimental Section

### 2.1. Materials

Polysulfone (PSf, Udel P-1700, Solvay Advanced Polymers, Alpharetta, GA, USA.) pellets were used as polymer for the fabrication of hollow fiber membranes. N-Methyl-2-pyrrolidone (NMP, purity > 99.5%, Merck, Darmstadt, Germany.) served as the solvent for preparing the polymer dope solution and was used without further purification. Silicon carbide nanoparticles (β-SiC; average particle size: 45–65 nm; purity: 99.9%; Nanoshel, London, UK) were used as the inorganic filler. 1H,1H,2H,2H-Perfluorodecyltriethoxysilane (PFDTES, 97%, Sigma-Aldrich, St. Louis, MO, USA) was used as the fluorinated silane coupling agent to surface-functionalize the SiC nanoparticles before their incorporation into the polymer dope solution for the fabrication of mixed-matrix hollow fiber membranes. Absolute ethanol (≥99.8%, Sigma-Aldrich) was used as the solvent during the fluorination procedure, while glacial acetic acid (≥99%, Sigma-Aldrich) and deionized (DI) water facilitated the hydrolysis and condensation reactions of the silane coupling agent. Tap water was employed as the non-solvent in the coagulation bath during the phase inversion process. For the CO_2_ absorption experiments, distilled water and aqueous sodium hydroxide (NaOH) solution were used as absorbents. A 1 M NaOH solution was prepared by dissolving analytical-grade sodium hydroxide pellets (≥98%, Sigma-Aldrich) in DI water. For the titration experiments, 0.05 M standardized hydrochloric acid (HCl) and sodium hydroxide (NaOH) solutions obtained from Sigma-Aldrich were employed. Carbon dioxide (CO_2_) with a purity of at least 99.99% was provided in a compressed gas cylinder and introduced directly into the experimental system without any further purification. Unless stated otherwise, all chemicals and reagents were utilized in their original form as supplied by the respective manufacturers.

### 2.2. Fluorination of SiC Nanoparticles

Surface fluorination of SiC nanoparticles was carried out using PFDTES as a fluorinated silane coupling agent. Initially, the required amount of SiC nanoparticles was dispersed in absolute ethanol and ultrasonicated for 30–45 min to disrupt particle agglomerates and obtain a stable, homogeneous suspension. The suspension was subsequently stirred mechanically while PFDTES was added dropwise at a dosage of 0.6 mL per gram of SiC. To initiate the hydrolysis of the triethoxysilane groups, a small amount of deionized DI water together with a few drops of glacial acetic acid was introduced into the suspension. Acetic acid acted as a mild acid catalyst, promoting the hydrolysis of the ethoxy groups (–OC_2_H_5_) to generate reactive silanol (Si–OH) species. Following reagent addition, the reaction mixture was maintained at 60 °C under continuous magnetic stirring for 6 h to facilitate both the hydrolysis of PFDTES and the subsequent condensation reactions between the silanol groups and hydroxyl functionalities naturally present on the SiC surface. This process resulted in the formation of stable Si–O–Si covalent bonds, thereby grafting fluorinated alkyl chains onto the particle surface. After completion of the reaction, the F-SiC nanoparticles were separated by centrifugation and washed several times with absolute ethanol followed by deionized water to remove physically adsorbed silane molecules and residual reaction by-products. The purified nanoparticles were subsequently dried in a vacuum oven at 60–80 °C for 12 h to eliminate residual solvents and moisture before being stored in a desiccator for subsequent membrane fabrication.

### 2.3. Preparation of Dope Solutions

Mixed matrix PSf hollow fiber membranes were fabricated by incorporating F-SiC nanoparticles into the polymer matrix using the wet phase inversion technique. F-SiC was introduced into the dope solution at concentrations of 0, 2, 4, and 6 wt.% (based on the total dope composition) to evaluate the influence of filler loading on the rheological behavior of the spinning dope and the resulting characteristics of the hollow fiber membranes. The membrane dope formulations containing 0, 2, 4, and 6 wt.% F-SiC were designated as S0, S2, S4, and S6, respectively, where the numerical suffix denotes the F-SiC loading (wt.%) relative to the weight of PSf. For each membrane composition, the designated amount of F-SiC was added to NMP and sonicated for about 30 min to disperse the nanoparticles effectively, prevent agglomeration, and form a homogeneous suspension. PSf pellets were then added gradually to the suspension under continuous magnetic stirring to facilitate complete polymer dissolution while maintaining uniform nanoparticle distribution throughout the solution. The resulting mixture was stirred at ambient temperature for 12–24 h until a uniform and highly viscous dope solution was obtained. To eliminate entrapped air generated during the mixing process, the dope solutions were subsequently degassed by ultrasonication for at least 1 h before spinning. This step was essential for producing a homogeneous spinning solution and minimizing the formation of structural defects, such as pinholes and macrovoids, in the hollow fiber membranes. The compositions of all dope formulations containing different F-SiC loadings, together with their corresponding polymer and solvent contents, are summarized in Table 1. The rheological properties of the prepared dope solutions were characterized by measuring their dynamic viscosity using a Brookfield digital viscometer (Cole-Parmer, Vernon Hills, IL, USA). Prior to each measurement, the solutions were mixed thoroughly and allowed to equilibrate to room temperature (25 ± 2 °C) to maintain uniform experimental conditions. A suitable spindle was then placed into the polymer solution, and the viscosity was measured once a stable reading had been reached. All measurements were performed in triplicate, and the reported viscosity represents the average of three independent determinations. The viscosity results were subsequently correlated with the F-SiC loading to assess the effect of nanoparticle incorporation on the rheological behavior and spinnability of the mixed matrix dope solutions.

### 2.4. Hollow Fiber Fabrication

Hollow fiber membranes based on PSf were prepared by employing the wet-phase inversion spinning method. The degassed polymer solution was then loaded into a stainless-steel reservoir and delivered to a laboratory-scale hollow fiber spinning unit fitted with a concentric spinneret. Simultaneously, a bore fluid composed of NMP and deionized water (90:10 wt.%) was injected through the inner orifice of the spinneret using a syringe pump, while the polymer dope was extruded through the outer annulus at a controlled flow rate. The flow rates of both the polymer solution and bore fluid were carefully adjusted to maintain a stable spinning process and to produce hollow fibers with uniform geometry and lumen dimensions. The freshly extruded fibers were introduced into a water coagulation bath without passing through an air gap. Eliminating the air gap promoted immediate solvent–non-solvent exchange, suppressing the formation of an excessively dense outer skin and facilitating the development of a highly porous membrane surface. As NMP diffused from the polymer solution into the coagulation bath while water simultaneously penetrated the nascent fibers, instantaneous liquid–liquid demixing occurred. Following spinning, the hollow fibers remained immersed in the deionized water bath for a minimum of 24 h to ensure complete phase inversion and thorough extraction of residual solvent. The membranes were then repeatedly washed with fresh deionized water to remove any remaining traces of NMP and other soluble impurities. Finally, the hollow fibers were suspended vertically and dried under ambient laboratory conditions before being used for membrane characterization, module fabrication, and CO_2_ absorption experiments. The operating conditions employed during the spinning process are summarized in Table 2.

### 2.5. Characterization of Hollow Fiber Membranes

The morphological characteristics of the PSf hollow fiber membranes were examined using a tabletop scanning electron microscope (SEM, Hitachi TM3000, Hitachi High-Tech Corporation, Tokyo, Japan). Both the membrane surface and cross-sectional morphologies were analyzed to investigate the influence of F-SiC nanoparticle incorporation on membrane formation during the phase inversion process. To obtain cross-sectional images, membrane specimens were cryo-fractured after immersion in liquid nitrogen, producing clean fracture surfaces without structural deformation. The fractured samples were mounted onto aluminum stubs using conductive carbon tape. Owing to the low-vacuum operating mode of the TM3000, a thin gold coating was deposited by sputter coating when required to minimize surface charging and improve image resolution. Surface observations were conducted on the outer selective layer, whereas membrane cross-section images provided information on the formation of the internal asymmetric structure. The elemental composition of the membrane surfaces was determined by energy-dispersive X-ray spectroscopy (EDX, Hitachi High-Tech Corporation, Tokyo, Japan.) coupled with the Hitachi TM3000 SEM. This analysis was performed to verify the successful incorporation of fluorinated SiC nanoparticles into the fabricated membranes. Membrane specimens were cut into small sections and fixed onto stubs using carbon tape before analysis. Measurements were carried out under vacuum using a suitable accelerating voltage to ensure sufficient X-ray excitation. Elemental spectra were obtained from selected representative areas of the membrane surface, while the corresponding concentrations of carbon (C), oxygen (O), sulfur (S), fluorine (F), and silicon (Si) were determined using the instrument’s built-in analytical software.

Surface roughness was characterized using AFM operating in tapping mode with a Bruker Dimension Icon (Bruker Corporation, Billerica, MA, USA). A silicon cantilever probe featuring a nominal tip radius of 5–10 nm and a resonance frequency in the range of approximately 200–350 kHz was utilized throughout the measurements, which were performed under ambient laboratory conditions. Before scanning, samples were trimmed to an appropriate size and secured onto a sample holder using double-sided adhesive tape. Images of the membrane surface were collected over scan areas of 5 × 5 µm^2^ and/or 10 × 10 µm^2^ to provide representative information on membrane morphology. To improve statistical reliability, several randomly selected locations were scanned for each membrane sample. The acquired height profiles were processed using the software to calculate the average surface roughness (Ra) and root-mean-square roughness (Rq), which were obtained from the deviation of surface heights relative to the mean reference plane.

The wettability of the hollow fiber membrane surfaces was assessed by measuring the static water contact angle with a Dataphysics OCA optical contact angle goniometer (Dataphysics, Filderstadt, Germany). Contact angle measurements were carried out under controlled room-temperature conditions. A droplet of deionized water (2–5 µL) was gently placed on the outer membrane surface using a microsyringe, and its profile was immediately captured by the instrument’s integrated high-resolution camera. Once the droplet had stabilized for several seconds, the static contact angle was determined by applying the Young–Laplace fitting approach available in the instrument software. At least five measurements were recorded at different locations on each membrane sample, and the average value was reported.

The resistance of the fabricated hollow fiber membranes to water penetration was characterized by determining the critical water entry pressure (CEPw), defined as the lowest transmembrane pressure at which water begins to enter the membrane pores and emerge from the outer surface. The same membrane module used for gas permeation experiments was employed for this test. Distilled water was introduced through the lumen side of the fibers, while the applied pressure was increased incrementally by 0.5 bar. After each pressure increase, the system was maintained at constant pressure for 30 min to observe any evidence of water breakthrough. The CEPw value was assigned as the pressure at which the first water droplet became visible on the external membrane surface.

The overall membrane porosity (ε) was determined using the gravimetric method, which estimates the ratio of pore volume to total membrane volume. Membrane porosity was calculated using Equation (1):

(1) $$\begin{array}{c} \varepsilon =\frac{(w_{1}-w_{2})/\rho_{w}}{(w_{1}-w_{2})/\rho_{w}+w_{2}/\rho_{p}} \end{array}$$

where ε represents membrane porosity (%), *w*_1_ is the weight of the wet membrane (g), *w*_2_ is the weight of the dry membrane (g), *ρw* is the density of water (g cm^−3^), and *ρp* is the density of the polymer (g cm^−3^). Five membrane samples were analyzed for porosity measurements. Prior to testing, the hollow fibers were immersed in tap water for three days to ensure complete solvent exchange and removal of residual solvent. The samples were subsequently soaked in distilled water for an additional 24 h to fully saturate the membrane pores. Excess water inside the fiber lumen was carefully removed using a gentle air stream before recording the wet weight. The membranes were then dried in a vacuum oven at 120 °C for 2 h, after which the dry weight was measured for porosity calculation.

Gas permeation tests were conducted to characterize the average pore diameter and effective surface porosity of the fabricated hollow fiber membranes. In each test, two hollow fibers were sealed at one end using epoxy resin, whereas their other ends were positioned inside a stainless-steel tube and firmly secured, providing an effective membrane length of approximately 10 cm. Nitrogen (N_2_) was used as the test gas because of its inert nature and predictable transport properties, enabling reliable evaluation of gas transport through membrane pores without interaction with the membrane material. During the permeation tests, N_2_ was supplied to the shell side of the hollow fiber module, and the operating pressure was controlled between 1 and 4 bar (gauge pressure) using a pressure regulator. The permeated gas collected from the lumen side was quantified by measuring its volumetric flow rate at ambient temperature using a soap-bubble flow meter.

The analysis of gas transport was based on the assumption that membrane pores possess cylindrical and straight geometries, and that gas movement occurs through the simultaneous contribution of Knudsen diffusion and viscous Poiseuille flow. Accordingly, the total permeance of permeated gas is:

(2) $$\bar{P}=P_{P}+P_{K}=\frac{2}{3}{\left(\frac{8RT}{\pi M}\right)}^{0.5}\frac{r_{p,m}}{RT}\frac{\varepsilon }{L_{p}}+\frac{1}{8\mu }\frac{r_{p,m}^{2}}{RT}\frac{\varepsilon }{L_{p}}\bar{p}$$

$$\bar{P}=A+B\bar{p}$$

In this equation, $\bar{P}$ represents the overall gas permeance (mol/m^2^·Pa·s), with *P_P_* and *P_K_* denoting the individual contributions from Poiseuille (viscous) flow and Knudsen diffusion, respectively (both in mol/m^2^·Pa·s). R is the universal gas constant (8.314 J/mol·K), and T denotes absolute temperature (K). M corresponds to the gas molecular weight (kg/mol), while *r*_*p*,*m*_ refers to the average pore radius (m). The symbol *μ* stands for gas viscosity (Pa·s), *ε* for surface porosity, *L_P_* for the effective length of the pores (m), and $\bar{p}$ for the mean pressure (Pa). By plotting $\bar{P}$ against $\bar{p}$, the resulting intercept (A) and slope (B) can then be used to determine the mean pore radius and effective surface porosity, as shown below.

(3) $$r_{p,m}=\frac{16}{3}\frac{B}{A}{\left(\frac{8RT}{\pi M}\right)}^{0.5}\mu$$

(4) $$\frac{\varepsilon }{L_{P}}=\frac{8\mu RTB}{r_{p,m}^{2}}$$

It is worth noting that several techniques exist for characterizing membrane pore size and pore size distribution, with the gas permeation test being just one option among them. Studies have shown that, despite yielding different absolute values, pore sizes obtained through these various methods tend to correlate with one another [24].

The mechanical robustness of the fabricated hollow fiber membranes was further evaluated through a collapse pressure test. The test was performed using the same gas permeation setup by gradually increasing the shell-side pressure of the membrane module in increments of $0.5\times 10^{5}$ Pa. The collapse pressure was identified as the pressure point at which a sudden variation in the lumen-side gas flow rate occurred, indicating the initiation of membrane deformation, pore structure distortion, or complete collapse of the hollow fiber structure.

### 2.6. CO_2_ Absorption Experiments

CO_2_ absorption tests were carried out using a laboratory-scale gas–liquid membrane contactor incorporating the fabricated PSf hollow fiber membranes. The detailed characteristics of the membrane contactor module are summarized in Table 3.

The membrane contactor consisted of ten hollow fibers housed within a cylindrical shell, thereby establishing a defined membrane surface area for gas–liquid mass transfer. The membrane acted as a physical barrier between the gas and liquid phases, enabling CO_2_ transport through the membrane pores while preventing direct mixing of the two streams. All absorption tests were carried out at steady state under room-temperature conditions. Two different liquid absorbents were employed: distilled water as a physical absorbent and a 1.0 M aqueous sodium hydroxide (NaOH) solution as a reactive absorbent. The NaOH solution was prepared by dissolving analytical-grade sodium hydroxide pellets in deionized water immediately before each experiment to minimize the influence of atmospheric CO_2_ absorption during storage. During operation, pure CO_2_ gas was introduced into the lumen side of the hollow fibers, whereas the liquid absorbent was circulated through the shell side of the module. The gas and liquid flow rates were controlled using calibrated flow meters and maintained at 100 mL min^−1^ and 50–300 mL min^−1^, respectively. The pressure of the gas stream was maintained at 1 bar, while the liquid-side pressure was adjusted to approximately 0.2 bar higher than the gas side to avoid gas penetration through the membrane and maintain a stable gas–liquid interface. Each experiment was operated for 30 min before sample collection to ensure that steady-state conditions had been achieved. All CO_2_ absorption flux measurements were performed in triplicate (N = 3) at each operating condition, and results are reported as mean ± standard deviation.

For the NaOH-based absorption system, CO_2_ removal occurred through an irreversible chemical reaction between dissolved CO_2_ and hydroxide ions, producing carbonate and bicarbonate species. In contrast, CO_2_ uptake in distilled water was governed mainly by physical dissolution and diffusion into the liquid phase. For the physical absorption experiments using distilled water, the concentration of absorbed CO_2_ in the liquid phase was determined by acid–base titration. The collected liquid samples were analyzed by titration against a standardized 0.05 M NaOH solution, with phenolphthalein serving as the indicator. The dissolved CO_2_ content was quantified based on the volume of NaOH solution consumed at the equivalence point, assuming that CO_2_ present in the aqueous phase was converted to bicarbonate ions. The absorbed CO_2_ quantity was calculated according to Equation (5):

(5) $$\begin{array}{c} n_{CO_{2}}=C_{NaOH}\times V_{NaOH} \end{array}$$

where $n_{CO_{2}}$ represents the amount of absorbed CO_2_ (mol), $C_{NaOH}$ is the NaOH concentration (mol L^−1^), and $V_{NaOH}$ is the volume of NaOH consumed during titration (L).

The corresponding CO_2_ flux was determined using Equation (6):

(6) $$\begin{array}{c} J_{CO_{2}}=\frac{n_{CO_{2}}}{At} \end{array}$$

where $J_{CO_{2}}$ is the CO_2_ flux (mol m^−2^ s^−1^), A is the effective membrane contact area (m^2^), and t is the absorption time (s).

For the chemical absorption experiments employing 1.0 M NaOH solution, CO_2_ removal occurred through the following reaction:

$$CO_{2}+2OH^{-}\to CO_{3}^{2-}+H_{2}O$$

The residual NaOH concentration after absorption was determined through back titration with a standardized hydrochloric acid (HCl) solution using phenolphthalein as the indicator. The amount of absorbed CO_2_ was calculated based on the stoichiometric consumption of hydroxide ions according to Equation (7):

(7) $$\begin{array}{c} n_{CO_{2}}=\frac{C_{NaOH}V_{NaOH}-C_{HCl}V_{HCl}}{2} \end{array}$$

where $n_{CO_{2}}$ is the absorbed CO_2_ amount (mol), $C_{NaOH}$ and $V_{NaOH}$ are the initial NaOH concentration (mol L^−1^) and volume (L), respectively, while $C_{HCl}$ and $V_{HCl}$ represent the HCl concentration (mol L^−1^) and titrant volume (L), respectively.

The CO_2_ flux for the reactive absorption system was calculated using Equation (8):

(8) $$\begin{array}{c} J_{CO_{2}}=\frac{n_{CO_{2}}}{At} \end{array}$$

where $J_{CO_{2}}$ denotes the CO_2_ flux (mol m^−2^ s^−1^), A is the effective membrane area (m^2^), and t is the operating time (s). The schematic representation of the experimental gas–liquid membrane contactor setup is provided in Figure 1.

To evaluate the long-term operational stability and resistance to pore wetting of the membrane contactors, continuous CO_2_ absorption experiments were conducted using distilled water as the absorbent under fixed operating conditions. Distilled water was selected to evaluate the intrinsic resistance of the membranes to pore wetting without the additional effects of prolonged alkaline exposure. The liquid and gas flow rates were maintained at 50 and 100 mL min^−1^, respectively, while the gas and liquid pressures were kept identical to those used in the short-term absorption experiments. The absorption system was operated continuously for 145 h. Liquid samples were collected from the absorbent outlet at approximately 5 h intervals throughout the test, and the absorbed CO_2_ concentration was determined by acid–base titration using standardized 0.05 M NaOH with phenolphthalein as the indicator, following the same procedure and calibration described above for the short-term physical-absorption experiments (Equation (5)). At each sampling interval, three replicate liquid samples were collected and analyzed (N = 3), and the corresponding CO_2_ absorption flux is reported as mean ± standard deviation. The corresponding CO_2_ absorption flux at each sampling time was calculated using Equation (6). The change in absorption flux with operating time was used to evaluate the long-term operational stability and resistance to pore wetting of the membranes under the tested conditions. Long-term stability testing with 1 M NaOH was not performed in this study; therefore, the present results do not directly establish the long-term resistance of the membranes to combined alkaline exposure and pore wetting. Extended testing with alkaline absorbents is considered an important subject for future investigation.

### 2.7. Determining Membrane Resistance to Mass Transfer

To quantify the resistance to mass transfer offered by the membrane itself, the Wilson plot approach can be applied [25]. When a hydrophobic hollow-fiber membrane with gas-filled pores is employed for CO_2_ absorption, the total resistance to mass transfer ($K_{O}^{-1}$) can be described using a series-resistance model [26]. When water is used for physical absorption and pure CO_2_ serves as the feed gas, resistance on the gas side becomes negligible, meaning the overall resistance can be attributed solely to the liquid-phase and the membrane. Resistance from the liquid phase scales with liquid velocity according to $V_{l}^{-\propto }$, with α representing an empirically determined constant and *V_l_* denoting the liquid velocity (m/s), obtained by dividing the liquid flow rate (m^3^/s) by the cross-sectional area available for liquid flow (m^2^)—this area being the module’s total cross-section minus the combined cross-sectional area occupied by the hollow fibers. Plotting $K_{O}^{-1}$ against $V_{l}^{-\propto }$ produces a linear relationship, referred to as the Wilson plot, where α is chosen to give the best linear fit to the experimental data. The y-intercept of this plot yields the membrane’s mass transfer resistance. The overall mass transfer coefficient itself is obtained from a mass balance taken along the hollow-fiber length [25]:

(9) $$K_{o}=-\frac{Q_{l}}{A_{i}}\ln\left(1-\frac{C_{l,o}}{HC_{g}}\right)$$

where *Ql* denotes the liquid flow rate (m^3^/s), *Ai* is the internal surface area of the hollow fibers (i.e., the gas–liquid contact area, m^2^), and *C_l,o_* and *C_g_* denote, respectively, the concentration of CO_2_ in the liquid outlet stream and on the gas side (mol/m^3^), and H refers to the Henry’s law constant, taken as 0.85 for the CO_2_–water system.

## 3. Results and Discussion

### 3.1. Morphological and Physicochemical Characterization of the Fabricated Membranes

The cross-sectional SEM micrographs of the pristine PSf and F-SiC modified hollow fiber membranes are illustrated in Figure 2. The pristine PSf membrane (0 wt.% F-SiC) displays a classic asymmetric cross-section characterized by long, fully developed, finger-like macrovoids extending from both the inner and outer skin layers, separated by a thin, transitional sponge-like layer in the center. This structure is highly indicative of instantaneous demixing, where the rapid mutual diffusion of the solvent and water (non-solvent) leads to immediate liquid–liquid demixing upon contact with the coagulation bath and bore fluid. Upon the incorporation of F-SiC nanoparticles, a clear morphological shift from a finger-like structure to a predominantly sponge-like structure is observed. At 2 wt.% and 4 wt.% F-SiC loadings, the finger-like macrovoids begin to shorten, narrow, and adopt a restricted teardrop shape. At the maximum loading of 6 wt.% F-SiC, the finger-like voids are almost completely suppressed, appearing only as shallow cavities near the membrane boundaries, while a dense, thick, and highly uniform sponge-like structure dominates the entire fiber wall cross-section. This transition can be comprehensively understood by analyzing the synergistic interplay between the rheological properties and the surface chemistry of the dope solutions. As shown in Table 1, the dynamic viscosity of the casting solutions rose significantly from 1887 mPa·s (0 wt.%) to 2273 mPa·s (2 wt.%), 2887 mPa·s (4 wt.%), and finally 3597 mPa·s (6 wt.%).

From a kinetic standpoint, this near-doubling of the dope viscosity slows down the mass transfer rates. High viscosity reduces the diffusion coefficients of both the solvent (leaving the membrane) and the non-solvent (entering the membrane) during the spinning process, as described by the Stokes–Einstein relation. This kinetic hindrance prevents the rapid localized penetration of water into the dope, which is a prerequisite for finger-like macrovoid propagation. Simultaneously, the thermodynamic effect introduced by the F-SiC nanoparticles further promotes this structural transition. The presence of highly hydrophobic fluorine functional groups on the SiC surfaces repels the incoming water molecules from the coagulation bath and the bore fluid. This thermodynamic resistance delays the onset of demixing (delayed demixing), allowing the polymer chains ample time to relax, entangle, and aggregate into a denser, mechanically superior sponge-like network before solidifying. Additionally, at higher nanoparticle loadings (6 wt.%), the physical presence of well-dispersed or locally clustered F-SiC nanoparticles acts as a tortuous barrier. These rigid nanoparticles block the straight-line flow paths of the non-solvent, breaking up the continuous growth of macrovoids and forcing the development of a highly interconnected, tortuous, and robust sponge-like sublayer.

The SEM micrographs of the inner lumen surfaces of the pristine PSf and F-SiC modified membranes are displayed in Figure 3. The pristine membrane (0 wt.%) exhibits a distinct, highly interconnected micro-porous surface texture. This open structure is attributed to the low viscosity of the neat dope (1887 mPa·s), which facilitates rapid solvent/non-solvent exchange and instantaneous demixing at the bore fluid interface during the initial contact phase. In contrast, the incorporation of F-SiC nanoparticles induces a significant structural tightening of the lumen surface. With the addition of 2 wt.% F-SiC, the inner surface transitions into a noticeably smoother, more homogenous, and dense skin layer, a state that is maintained through the 4 wt.% and 6 wt.% loadings. Kinetically, the drastic elevation in dope viscosity (reaching up to 3597 mPa·s at 6 wt.%) heavily penalizes the mutual diffusion rates of the solvent and the bore fluid. This kinetic hindrance, complemented by the thermodynamic repulsion of water by the hydrophobic fluorinated functional groups on the SiC, induces a delayed demixing pathway. As a result, the polymer phase consolidates into a more uniform, compact, and low-porosity boundary layer before vitrification is finalized. Furthermore, starting from 2 wt.% and peaking distinctly at 6 wt.% F-SiC, a uniform distribution of bright, granular sub-micron features is observed across the membrane surfaces. Due to the higher material electron density of the inorganic silicon carbide relative to the PSf matrix, these features represent the embedded F-SiC nanoparticles protruding slightly from the skin layer. The highly uniform distribution and lack of severe, large-scale clusters—even at the maximum loading of 6 wt.%—demonstrates excellent compatibility between the fluorinated nanoparticles and the host polymer matrix, confirming the successful fabrication of well-dispersed mixed matrix hollow fibers.

The SEM micrographs capturing the outer skin morphology of the pristine PSf and F-SiC modified hollow fiber membranes are shown in Figure 4. The pristine membrane (0 wt.%) exhibits a highly textured surface with visible micro-porous architecture. Under a wet-spinning configuration (air gap = 0 cm), the nascent fiber is subjected to immediate immersion into the tap water coagulation bath upon leaving the spinneret die. This instant contact induces rapid, instantaneous liquid–liquid demixing at the outermost boundary, yielding the observed open surface texture. Conversely, the addition of F-SiC nanoparticles promotes a severe structural densification of the outer layer. Starting at 2 wt.% and proceeding through to 6 wt.%, the open micro-porous network completely disappears, replaced by a highly uniform, smooth, and dense selective skin layer. With solvent evaporation entirely eliminated by the zero-air-gap condition, this structural transition is driven by the rheological and thermodynamic behavior of the dopes. The steep increase in dynamic viscosity from 1887 mPa·s (0 wt.%) to 3597 mPa·s (6 wt.%) creates a formidable kinetic barrier that restricts the mass transfer rates between the solvent and the tap water non-solvent. Concurrently, the hydrophobic fluorine functionalities on the SiC repel the incoming water fronts, driving a localized delayed demixing mechanism. This prolonged precipitation interval allows the surface polymer chains to compact neatly before complete vitrification. Furthermore, the modified membranes (2–6 wt.%) present a growing population of well-dispersed, sub-micron bright nodes spread uniformly across the outer surface. These features correspond to the embedded F-SiC nanoparticles, which exhibit higher electron reflectivity than the host polymer matrix due to material density contrast. The excellent, isolated distribution of these nodes at higher loadings (4 wt.% and 6 wt.%) verifies that the fluorinated surface modification successfully hindered particle agglomeration, establishing a well-compatibilized mixed matrix structure at the selective skin layer.

To quantitatively evaluate the surface topography of the fabricated membranes, 2D AFM scans were performed on the outer surface of the hollow fibers, as illustrated in Figure 5. The corresponding average roughness (*R_a_*), root-mean-square roughness (*R_q_*), and maximum peak-to-valley height (*R_t_*) values are summarized in the inset of each scan. The pristine PSf membrane (0 wt.%) exhibits the highest surface roughness, with a *R_a_* of 42.92 nm and a high *R_t_* of 485.2 nm. The topography scan reveals a highly irregular, deeply textured surface featuring wide, dark depressions. Under zero-air-gap wet-spinning conditions, the immediate contact of the low-viscosity neat dope (1887 mPa·s) with tap water induces rapid liquid–liquid demixing. This instantaneous phase separation allows water to penetrate deep into local areas, yielding large, open surface micro-pores that are registered as distinct valleys by the AFM cantilever tip. Upon the incorporation of F-SiC nanoparticles, a dramatic decline in surface roughness is observed. For the 2 wt.% and 4 wt.% F-SiC modified membranes, *R_a_* drops sharply to 9.67 nm and 7.35 nm, respectively. This over 80% reduction in roughness is directly linked to the rheological and thermodynamic behavior of the modified dopes. The elevated viscosities (up to 2887 mPa·s) and hydrophobic fluorine groups delay the demixing rate at the interface of the tap water bath. This delayed demixing grants the polymer chains sufficient relaxation time to consolidate into a highly uniform, compact, and pore-free outer skin layer, effectively eliminating the deep topographical valleys. Notably, the pronounced reduction in surface roughness from S0 to S4 occurs concurrently with an increase, rather than a decrease, in water contact angle. This trend is inconsistent with roughness-amplification (Wenzel/Cassie–Baxter) behavior, which would predict that a smoother surface should move the contact angle of an intrinsically hydrophilic base polymer closer to, not further from, its flat-surface value. The observed increase in contact angle despite decreasing roughness therefore indicates that the change in surface chemistry—specifically, the increasing density of low-surface-energy fluorinated functional groups introduced by the F-SiC nanoparticles at the membrane surface—rather than surface topography, is the dominant factor governing the wettability trend across the sample series. Interestingly, at 6 wt.% F-SiC loading, the surface roughness increases slightly to a *R_a_* of 12.12 nm (*R_q_* = 15.64 nm). Rather than signifying polymer surface defects, this subtle upward trend is attributed to the “nano-protrusion effect” of the inorganic phase. At high loadings, the dense population of rigid, sub-micron F-SiC nanoparticles embedded close to the outer skin layer creates localized nanoscale peaks, visible as sharp, bright white features scattered across the AFM scan. However, due to the excellent compatibility and fine dispersion of the fluorinated nanoparticles, these protrusions remain extremely small and isolated. Consequently, the outer surface of the 6 wt.% membrane remains significantly smoother than that of the pristine control. While reduced surface roughness is generally associated with lower fouling propensity in membrane processes, this was not directly evaluated in the present study, and we do not draw a fouling-resistance conclusion from the AFM data alone.

The elemental composition of the pristine PSf and F-SiC-modified mixed-matrix hollow fiber membranes was analyzed by Energy Dispersive X-ray Spectroscopy (EDX), and the results are summarized in Table 4. EDX provides semi-quantitative elemental information from the analyzed membrane region. Therefore, the values reported in Table 4 represent the detected elemental mass fractions and should not be considered as bulk elemental compositions or stoichiometric ratios. The pristine membrane (0 wt.% F-SiC) showed only Carbon (C, 77.20 wt.%), Oxygen (O, 15.30 wt.%), and Sulfur (S, 7.50 wt.%), which are the major elements expected from the polysulfone matrix. No detectable Si or F was observed in the pristine membrane. After incorporation of the fluorinated SiC nanoparticles, Si and F signals were detected in the modified membranes. At 2 wt.% F-SiC loading, the detected Si and F contents were 1.10 and 0.80 wt.%, respectively. These values increased with increasing nominal F-SiC loading and reached 3.60 wt.% Si and 2.70 wt.% F at 6 wt.% loading. This increasing trend is consistent with the incorporation of the F-SiC filler into the PSf matrix and supports the presence of the modified nanoparticles in the analyzed membrane region. A gradual decrease in the relative contents of the major polymer elements was also observed with increasing F-SiC loading. For example, the S content decreased from 7.50 wt.% for the pristine membrane to 6.80 wt.% at 6 wt.% F-SiC loading. This change can be attributed to the increasing contribution of the inorganic filler to the detected elemental composition, which consequently reduces the relative contribution of the PSf matrix.

The physical, structural, wetting, and mechanical properties of the fabricated hollow fiber membranes are summarized in Table 5. The water contact angle increases progressively from 77.50 ± 2° for the pristine PSf membrane to 86 ± 1°, 91.60 ± 2°, and 107.0 ± 3° for the S2, S4, and S6 membranes, respectively, confirming the successful incorporation of F-SiC nanoparticles and the progressive reduction in membrane surface energy. The fluorinated functional groups introduced by PFDTES together with the homogeneous distribution of F-SiC nanoparticles effectively enhance the hydrophobicity of the membrane surface, thereby improving its resistance to pore wetting.

The CEPw also increases continuously with increasing F-SiC loading, rising from 4.00 ± 0.50 bar for the pristine membrane to 6.50 ± 0.50, 9.50 ± 0.50, and 10.00 ± 0.50 bar for the S2, S4, and S6 membranes, respectively. This improvement results from the synergistic effect of increasing surface hydrophobicity and decreasing pore size. According to the Young–Laplace equation [27,28] ($∆P=\frac{-2\gamma LCOS\theta }{r}$), both a higher water contact angle and a smaller pore radius increase the capillary pressure required for water penetration. Consequently, the continuous reduction in average pore size together with the enhanced hydrophobicity significantly improves the wetting resistance of the mixed -matrix membranes, making them more suitable for long-term gas–liquid membrane contactor operation.

A direct quantitative comparison using the Young–Laplace equation (γL = 72.8 mN/m, water, 25 °C), the measured contact angles, and the mean pore radii in Table 5 predicts ΔP ≤ 0 for S0 and S2 (θ < 90°, implying spontaneous wetting) and ΔP ≈ 0.3 and 5.5 bar for S4 and S6, respectively—values substantially lower than the measured CEPw of 4.0, 6.5, 9.5, and 10.0 bar for S0, S2, S4, and S6. This systematic underprediction, which is largest for S0 and diminishes with increasing F-SiC loading, reflects two well-recognized limitations of applying the classical Young–Laplace equation to microporous hollow fiber membranes. First, the contact angle used is the macroscopic sessile-drop value measured on a flat surface, whereas water breakthrough is governed by the local, effective contact angle at the curved, rough pore-mouth geometry; surface roughness (highest for S0, Table 5) is known to raise this local resistance to liquid entry above what the bulk contact angle predicts. Second, the pore radius in Table 5 is a mean transport radius derived from the gas-permeation model (Equations (3) and (4)) and integrates contributions across the full pore-size distribution, whereas CEPw is physically controlled by the largest, most easily wetted pore rather than the population-averaged radius; substituting the mean radius for the pore-entry-controlling radius therefore systematically underpredicts the true breakthrough pressure. Both effects are well documented in the membrane-wetting literature, and accordingly the Young–Laplace relation is used here to rationalize the qualitative trend of increasing CEPw with contact angle and decreasing pore size, while the measured CEPw values (Table 5) are taken as the definitive wetting-resistance metric for this study.

The average pore size decreases from 161.67 nm for S0 to 155.22 nm for S2, 138.33 nm for S4, and 77.96 nm for S6. The reduction becomes particularly pronounced at the highest F-SiC loading, indicating substantial densification of the membrane structure at 6 wt.% F-SiC. This trend is consistent with the increasing viscosity of the spinning dope and the morphological changes observed by SEM. The viscosity increases from 1887 ± 35 mPa·s for S0 to 3597 ± 91 mPa·s for S6, which can slow solvent/non-solvent exchange during phase inversion and restrict the development of larger pore structures. Accordingly, the SEM images show a progressive suppression of finger-like macrovoids and development of a more compact sponge-like morphology with increasing F-SiC loading. The pronounced reduction in average pore size for S6 is therefore consistent with the highly compact morphology observed at the highest nanoparticle concentration.

In contrast, the effective surface porosity does not follow the same monotonic trend as the average pore size. The ε/Lp value increases from 17.66 m^−1^ for S0 to 29.91 m^−1^ for S2 and reaches a maximum of 40.54 m^−1^ for S4, followed by a decrease to 29.75 m^−1^ for S6. This non-monotonic behavior suggests that the effective surface porosity is influenced not only by the overall membrane void fraction but also by the accessibility and characteristics of the gas-transport pathways. The increase from S0 to S4 may indicate improved effective gas-accessible pathways associated with the modified pore structure, whereas the subsequent decrease for S6 is consistent with the stronger structural densification and pronounced reduction in pore size at the highest F-SiC loading. Thus, the results indicate that intermediate F-SiC loading produces a different balance between pore size and effective surface porosity than the highly compact S6 structure.

The overall porosity of the hollow fiber membranes decreases progressively with increasing F-SiC loading, declining from 78.5 ± 2% for the pristine membrane to 74.1 ± 2%, 70.6 ± 2%, and 69.3 ± 2% for the S2, S4, and S6 membranes, respectively. This continuous reduction is attributed to the significant increase in dope solution viscosity following the incorporation of F-SiC nanoparticles, which slows the solvent/non-solvent exchange during phase inversion and promotes delayed demixing. Consequently, the rapid formation of finger-like macrovoids is progressively suppressed, leading to the development of a dense sponge-like structure with a lower void fraction, as confirmed by the SEM cross-sectional images. Furthermore, the uniformly dispersed rigid nanoparticles restrict polymer chain mobility and hinder pore development, producing a more compact membrane matrix. The continuous decrease in overall porosity is in excellent agreement with the simultaneous reductions in average pore size and effective surface porosity, confirming that increasing F-SiC loading promotes progressive densification of both the membrane surface and internal structure. This compact morphology is expected to enhance wetting resistance and mechanical stability, which are desirable characteristics for long-term operation in gas–liquid membrane contactors. The improvement in wetting resistance follows directly from the Young–Laplace relationship discussed above: as macrovoids are suppressed and the average pore size decreases, the critical liquid-entry pressure required to intrude the pore network increases, since this pressure scales inversely with pore radius. A denser, sponge-like structure therefore requires a higher transmembrane pressure before the liquid absorbent can penetrate and wet the pores—a key failure mode that otherwise floods the gas-transport pathway and sharply reduces mass-transfer performance. The gain in mechanical stability arises from a complementary structural effect: macrovoids act as stress concentrators and reduce the load-bearing polymer fraction per unit cross-section, so their suppression, together with the restricted chain mobility imparted by the uniformly dispersed F-SiC nanoparticles, increases the effective solid fraction and removes these weak points from the matrix. This is consistent with the collapsing-pressure results reported below, which rise monotonically with F-SiC loading. Because gas–liquid membrane contactors are typically operated with the liquid phase held at a slightly higher pressure than the gas phase to prevent gas bubble-through, the membrane must simultaneously resist liquid intrusion and withstand this pressure differential without structural deformation; the densified morphology produced by F-SiC incorporation addresses both requirements through the same underlying structural change.

The collapsing pressure increases continuously from 4.00 × 10^5^ Pa for the pristine membrane to 8.50 × 10^5^, 10.50 × 10^5^, and 11.00 × 10^5^ Pa for the S2, S4, and S6 membranes, respectively, demonstrating that incorporation of F-SiC nanoparticles substantially enhances the mechanical stability of the hollow fibers. The improved mechanical strength is attributed to two complementary factors. First, the transition from a macrovoid-rich structure to a dense sponge-like morphology provides a more uniform distribution of external stress throughout the membrane wall. Second, the rigid SiC nanoparticles act as reinforcing fillers that restrict polymer chain mobility and improve load transfer within the polysulfone matrix. Consequently, the S6 membrane exhibits the highest mechanical strength while simultaneously possessing the smallest pore size, lowest effective surface porosity, highest hydrophobicity, and highest liquid entry pressure, confirming the beneficial effect of F-SiC nanoparticles on both structural integrity and wetting resistance.

Figure 6 presents the N_2_ permeance of the fabricated hollow fiber membranes as a function of mean transmembrane pressure. For all membranes, the N_2_ permeance increases with increasing mean transmembrane pressure, indicating pressure-dependent gas transport through the membrane pore structure. However, the overall N_2_ permeance decreases with increasing F-SiC loading, with the most pronounced reduction observed for S6. This behavior is consistent with the changes in the structural parameters calculated from the N_2_ permeation data and summarized in Table 5.

The average pore size decreases progressively from 161.67 nm for S0 to 155.22 nm, 138.33 nm, and 77.96 nm for S2, S4, and S6, respectively. The substantial reduction in pore size, particularly for S6, restricts the available pathways for N_2_ transport and consequently contributes to the lower gas permeance. The effective surface porosity (ε/Lp), which represents the effective contribution of accessible pores to gas transport, shows a different trend. It increases from 17.66 m^−1^ for S0 to 29.91 m^−1^ for S2 and reaches a maximum of 40.54 m^−1^ for S4, before decreasing to 29.75 m^−1^ for S6. Thus, the N_2_ permeance is governed by the combined effects of pore size and effective surface porosity rather than by either parameter alone.

The relatively high N_2_ permeance of S2 and particularly S4 can be related to their higher effective surface porosity compared with S0, which provides a greater effective contribution of accessible gas-transport pathways. Although the average pore size decreases from S0 to S4, the increase in effective surface porosity partially compensates for the reduction in pore size, resulting in comparatively favorable gas transport. In contrast, the sharp decrease in both average pore size and effective surface porosity for S6 results in a more pronounced restriction of N_2_ transport and explains its lower permeance.

These changes are also consistent with the membrane morphology observed by SEM. The incorporation of F-SiC increases the viscosity of the spinning dope from 1887 ± 35 mPa·s for S0 to 2273 ± 35, 2887 ± 65, and 3597 ± 91 mPa·s for S2, S4, and S6, respectively. The increased viscosity can retard solvent/non-solvent exchange during phase inversion and progressively suppress the formation of large pore structures. Accordingly, the SEM images show a transition from well-developed finger-like macrovoids in S0 toward a more compact sponge-like morphology with increasing F-SiC loading, with the most pronounced structural densification observed for S6. Therefore, the reduction in N_2_ permeance with increasing F-SiC loading, particularly at S6, can be reasonably associated with the combined reduction in pore size, changes in effective surface porosity, and progressive structural densification of the membrane. It should be noted that the linear regressions used to derive the intercept (A) and slope (B) from the N_2_ permeance data (Figure 6) yielded R^2^ values of 0.89, 0.84, 0.98, and 0.81 for S0, S2, S4, and S6, respectively. The comparatively lower R^2^ values obtained for S0, S2, and S6 (0.81–0.89) indicate greater scatter in the underlying permeance data for these membranes and introduce correspondingly greater uncertainty into the derived pore size and ε/Lp values in Table 5. The S4 membrane, by contrast, showed the strongest linear fit (R^2^ = 0.98), so its derived structural parameters can be regarded as the most reliable among the series. While the overall trends in pore size and effective surface porosity with F-SiC loading are consistent with the SEM and CEPw data and are therefore considered robust, the absolute pore size and ε/Lp values for S0, S2, and S6 should be interpreted with appropriate caution given the fit quality.

### 3.2. CO_2_ Absorption Performance

Figure 7 illustrates the influence of distilled water flow rate on the CO_2_ absorption flux of the pristine PSf membrane and the F-SiC mixed -matrix membranes. For all membranes, the CO_2_ absorption flux increases continuously with increasing liquid flow rate from 50 to 300 mL min^−1^, indicating that higher liquid velocities reduce the liquid-side mass-transfer resistance by decreasing the thickness of the boundary layer surrounding the membrane surface. Consequently, the driving force for CO_2_ transport is enhanced, resulting in higher absorption rates. The incorporation of F-SiC nanoparticles substantially improves the CO_2_ absorption performance compared with the pristine membrane. The pristine PSf membrane (S0) exhibits the lowest performance, with the CO_2_ flux increasing from 9.9 × 10^−5^ mol m^−2^ s^−1^ at 50 mL min^−1^ to 4.2 × 10^−4^ mol m^−2^ s^−1^ at 300 mL min^−1^. The S2 membrane shows higher absorption fluxes ranging from 2.7 × 10^−4^ to 5.9 × 10^−4^ mol m^−2^ s^−1^ over the same flow-rate range.

Among all fabricated membranes, the S4 membrane exhibits the highest CO_2_ absorption performance. Its absorption flux increases from 4.0 × 10^−4^ mol m^−2^ s^−1^ at 50 mL min^−1^ to 7.6 × 10^−4^ mol m^−2^ s^−1^ at 300 mL min^−1^, representing approximately a 90% enhancement compared with the pristine membrane at the highest liquid flow rate. This superior performance can be attributed to the optimum balance between membrane hydrophobicity, pore structure, and gas permeability. As summarized in Table 5, the S4 membrane possesses a contact angle of 91.6°, a CEPw of 9.5 bar, and an average pore size of 138.33 nm, providing high resistance to pore wetting while maintaining sufficient gas transport.

Although the S6 membrane exhibits the highest hydrophobicity (contact angle 107°) and the highest CEPw (10 bar), its CO_2_ absorption flux is slightly lower than that of S4, increasing from 3.7 × 10^−4^ to 7.3 × 10^−4^ mol m^−2^ s^−1^. The reduced performance is attributed to excessive membrane densification caused by the high nanoparticle loading, which decreases the average pore size to 77.96 nm and reduces the effective surface porosity to 29.75 m^−1^, thereby increasing the membrane mass-transfer resistance. These results demonstrate that an intermediate F-SiC loading (4 wt.%) provides the optimum membrane structure for physical CO_2_ absorption under the short-term flux conditions tested here [17].

Figure 8 presents the effect of 1 M NaOH flow rate on the CO_2_ absorption flux through the fabricated hollow fiber membranes. Similar to the distilled water system, increasing the absorbent flow rate significantly enhances CO_2_ absorption for all membranes. However, the absorption fluxes obtained with NaOH are substantially higher because CO_2_ is consumed through an irreversible chemical reaction with hydroxide ions, maintaining a much larger concentration driving force across the membrane. The pristine PSf membrane exhibits CO_2_ absorption fluxes ranging from 1.2 × 10^−4^ mol m^−2^ s^−1^ at 50 mL min^−1^ to 7.0 × 10^−4^ mol m^−2^ s^−1^ at 300 mL min^−1^. The S2 membrane shows a marked improvement, increasing from 2.8 × 10^−4^ to 9.9 × 10^−4^ mol m^−2^ s^−1^, corresponding to an increase of approximately 41% over the pristine membrane at the highest liquid flow rate.

The highest CO_2_ absorption performance is achieved by the S4 membrane, where the absorption flux increases continuously from 7.2 × 10^−4^ mol m^−2^ s^−1^ at 50 mL min^−1^ to 1.59 × 10^−3^ mol m^−2^ s^−1^ at 300 mL min^−1^. This value is approximately 2.3 times greater than that of the pristine membrane under identical operating conditions. The outstanding performance of S4 results from its favorable combination of improved membrane hydrophobicity, high wetting resistance, and moderate pore size, which minimizes pore wetting while maintaining efficient gas transport.

Although the S6 membrane possesses the highest hydrophobicity and wetting resistance, its maximum CO_2_ flux (1.55 × 10^−3^ mol m^−2^ s^−1^) is slightly lower than that of S4. The excessive reduction in pore size and effective surface porosity limits gas diffusion through the membrane, partially offsetting the benefits of enhanced hydrophobicity. Therefore, while increasing F-SiC loading improves membrane wetting resistance, excessive loading introduces additional membrane resistance that restricts overall mass transfer. These findings indicate that the S4 membrane provides the optimal balance between membrane permeability and wetting resistance for reactive CO_2_ absorption.

Figure 9 presents the Wilson plots used to evaluate the membrane mass-transfer resistance during CO_2_ absorption [29]. The best linear correlation was obtained using an exponent of −0.93 for *V*. This same form of relationship (1/K_o_ plotted against V^−0.93^) was previously employed by Yang and Cussler [30] to characterize liquid flow on the shell side of a hollow fiber membrane module. The slopes and intercepts obtained from the linear regression provide information on the velocity-dependent and velocity-independent contributions to the overall mass-transfer resistance, respectively.

The variation in the Wilson-plot slopes with F-SiC loading indicates that incorporation of the nanoparticles influences the liquid-side mass-transfer behavior of the membrane contactors. The changes in slope can be associated with modifications in the hydrodynamic and interfacial characteristics of the membrane module resulting from the changes in membrane morphology and surface properties. Therefore, the slope is considered a parameter describing the dependence of the liquid-side mass-transfer contribution on liquid velocity rather than a direct measure of the intrinsic membrane resistance. The intercept, representing the velocity-independent contribution to the overall mass-transfer resistance, also varies with F-SiC loading. This behavior indicates that modification of the membrane structure affects the resistance components that are not strongly dependent on liquid velocity. The changes in this contribution can be related to the combined effects of membrane morphology, pore structure, surface hydrophobicity, and gas accessibility. As discussed previously, F-SiC incorporation progressively changes the membrane morphology from well-developed finger-like macrovoids toward a more compact sponge-like structure, while also reducing the average pore size. These structural changes can influence the resistance to CO_2_ transport through the membrane.

The effect of F-SiC loading on the Wilson-plot parameters is particularly related to the balance between pore accessibility and membrane densification. At low and intermediate F-SiC loadings, modification of the membrane structure and surface characteristics can preserve favorable gas-transport pathways and reduce the susceptibility of the pores to liquid penetration. The increased hydrophobicity of the modified membranes is expected to contribute to maintaining gas-filled pores and a stable gas–liquid interfacial region. At the highest F-SiC loading, however, the substantially increased dope viscosity promotes a denser membrane morphology, accompanied by a marked reduction in average pore size and effective surface porosity. Such structural densification can increase the resistance to CO_2_ transport despite the enhanced hydrophobicity of the membrane. The Wilson-plot results therefore demonstrate that F-SiC incorporation affects both the velocity-dependent liquid-side contribution and the velocity-independent resistance components of the CO_2_ mass-transfer process. The observed changes are consistent with the independent membrane characterization results, including N_2_ permeance, average pore size, effective surface porosity, surface hydrophobicity, and SEM morphology. The combination of these effects determines the overall mass-transfer behavior of the hollow fiber membrane contactors and explains the dependence of CO_2_ absorption performance on F-SiC loading.

It is important to distinguish the intrinsic dry-membrane transport resistance—set by the pore size and structural parameter (ε/Lp) and reflected in the N_2_ permeance data (Section 3.1)—from the operational mass-transfer resistance obtained from the Wilson-plot intercept during CO_2_ absorption. These are not the same quantity. The dry-state data indicate that F-SiC incorporation increases the intrinsic resistance to gas transport through the pore structure itself, since both pore size and N_2_ permeance decrease with loading. The lower total operational resistance observed for S4 under wet operating conditions is therefore not attributable to a more open or more permeable dry pore structure. Rather, as Table 6 shows, it is driven predominantly by a substantially smaller velocity-dependent (liquid-film) contribution captured in the Wilson-plot slope (238.8 for S4 versus 1653.0 for S0); the velocity-independent membrane term (regression intercept) is essentially compa-rable between the two membranes (41,120 s/m for S4 versus 37,956 s/m for S0) and does not by itself explain the improvement. This pattern is consistent with suppression of pore wetting: the higher contact angle and CEPw of S4 raise the capillary entry pressure for the aqueous phase, so a larger fraction of the pore volume remains gas-filled during operation than in the pristine membrane, avoiding the additional liquid-phase diffusion resistance that arises once pores are wetted. We note that, in principle, the liquid-film (slope) term is expected to depend primarily on liquid hydrodynamics and module geometry, both held constant across membranes; the pore-wetting explanation for the ~7-fold reduction in slope from S0 to S4 is therefore an interpretation consistent with the CEPw, contact-angle, and long-term stability trends, rather than a mechanism isolated by a dedicated experiment, and should be treated as such. This interpretation is supported directly by the long-term stability data (Figure 10), where the onset of flux decline—attributed to progressive wetting—is delayed nearly fourfold (35 h to 125 h) and the cumulative flux loss at 145 h falls from 44.44% to 12.25% for S4 relative to S0. Because this resistance increase develops over time as wetting progresses, rather than being present from the start of the experiment, it confirms that the lower resistance of S4 reflects preserved gas-filled pore pathways rather than an intrinsically more open dry structure. At the highest loading (S6), the pore size and ε/Lp reduction become severe enough that this intrinsic-resistance penalty outweighs the wetting-suppression benefit, explaining why S6 does not outperform S4 despite its higher CEPw and contact angle.

Table 6 presents the Wilson-plot slope and intercept values for each membrane, separating the liquid-film and membrane contributions to the total mass-transfer resistance. Each regression was based on n = 10 data points per membrane, corresponding to the liquid flow rates tested (50–300 mL min^−1^). The lower R^2^ obtained for S2 (0.780) relative to S0, S4, and S6 (0.844–0.946) indicates greater uncertainty in its resolved slope and intercept, and comparisons involving S2’s resistance components should be interpreted accordingly. The slope (liquid-film resistance coefficient) and intercept (membrane resistance) were obtained from the linear regression of the Wilson plots (1/K_0_ vs. V^−0^·^93^, Figure 9) for each membrane. Gas-phase resistance is not tabulated, as it is negligible by design under the pure-CO_2_/distilled-water conditions used for the Wilson-plot measurements. Liquid-film resistance is additionally evaluated at the reference liquid flow rate of 50 mL min^−1^, matching the standard operating condition used elsewhere in this study.

Figure 10 illustrates the long-term CO_2_ absorption performance of the pristine PSf (S0) and fluorinated SiC-modified PSf (S4) hollow fiber membrane contactors using distilled water as the absorbent under constant operating conditions (liquid flow rate of 50 mL min^−1^ and gas flow rate of 100 mL min^−1^). S0 and S4 were selected for this test as the unmodified control membrane and the best-performing membrane identified from the short-term CO_2_ absorption experiments, respectively, in order to establish whether the optimum short-term performance of S4 translates into a durability advantage over the pristine baseline. As shown in the figure, the S0 membrane exhibited the first noticeable decline in CO_2_ absorption flux after only 35 h of continuous operation, whereas the S4 membrane maintained a nearly constant flux until approximately 125 h, indicating a substantial delay in the onset of membrane wetting. The initial CO_2_ absorption flux of the S0 membrane was 9.9 × 10^−5^ mol m^−2^ s^−1^, which progressively decreased to 5.5 × 10^−5^ mol m^−2^ s^−1^ after 145 h, corresponding to an 44.44% reduction in absorption performance. This rapid deterioration indicates progressive pore wetting, which increased the membrane mass-transfer resistance and reduced the effective gas–liquid interfacial area available for CO_2_ transport.

In contrast, the S4 membrane maintained a stable CO_2_ absorption flux for most of the operating period, with no appreciable decline observed until 125 h. Even after 145 h of continuous operation, the absorption flux decreased only slightly from 4.00 × 10^−4^ to 3.51 × 10^−4^ mol m^−2^ s^−1^, representing only a 12.25% reduction in flux. The delayed onset of performance decline and the minimal flux loss demonstrate the excellent anti-wetting characteristics and operational stability of the fluorinated SiC-modified membrane relative to the pristine control. The superior long-term performance of the S4 membrane is attributed to the incorporation of F-SiC nanoparticles, which enhanced membrane hydrophobicity, increased the liquid entry pressure, and preserved an interconnected porous structure for efficient gas transport. These characteristics effectively suppressed liquid penetration into the membrane pores, thereby maintaining gas-filled pathways and minimizing the increase in membrane mass-transfer resistance during prolonged operation [7]. The excellent stability of the S4 membrane is consistent with its higher water contact angle, greater CEPw, and lower total mass-transfer resistance obtained from the Wilson plot analysis, confirming that F-SiC modification effectively mitigates membrane wetting and ensures sustained CO_2_ absorption performance during extended operation. As this comparison was limited to the pristine and optimum membranes, the present long-term results should be interpreted as demonstrating the durability advantage of S4 over the unmodified S0 membrane rather than as establishing a complete loading-dependent trend in wetting resistance across all F-SiC contents. Long-term testing of the intermediate (S2) and highest-loading (S6) membranes is recommended in future work to confirm whether the trade-off between wetting resistance and mass-transfer resistance inferred from the short-term and structural data (Section 3.1 and Section 3.2) is also observed under extended operation.

Table 7 compares the membrane materials, modification strategies, wetting properties, and gas absorption performances reported in previous membrane contactor studies with those obtained in the present work. Previous investigations have mainly focused on incorporating inorganic fillers such as montmorillonite (MMT), silica, carbon nanotubes, Cu@4A composites, and metal oxides to improve membrane hydrophobicity and gas absorption performance. Although several studies reported higher CO_2_ absorption fluxes, these improvements were frequently achieved using different membrane configurations (e.g., flat-sheet membranes), nanofluid absorbents, or substantially different operating conditions, making direct quantitative comparison difficult. In addition, many previous studies emphasized either membrane hydrophobicity or gas absorption performance without simultaneously evaluating membrane wetting resistance, mechanical stability, and membrane mass-transfer resistance.

In contrast, the present study demonstrates that F-SiC nanoparticles effectively enhance multiple membrane characteristics simultaneously. The optimized S4 membrane achieved a water contact angle of 91.6°, a high CEPw of 9.5 bar (10 bar for S6), and maximum CO_2_ absorption fluxes of 7.6 × 10^−4^ mol m^−2^ s^−1^ using distilled water and 1.59 × 10^−3^ mol m^−2^ s^−1^ using 1 M NaOH. More importantly, the Wilson plot analysis confirmed that the optimized membrane possessed the lowest total operational mass-transfer resistance, demonstrating that F-SiC incorporation provides an optimal balance between hydrophobicity, pore structure, gas permeability, and wetting resistance. Unlike many previous studies that focused solely on increasing surface hydrophobicity or absorption flux, the present work provides a comprehensive evaluation of membrane morphology, mechanical strength, wetting resistance, gas transport characteristics, and mass-transfer behavior, highlighting the suitability of fluorinated SiC/PSf hollow fiber membranes for stable and efficient long-term CO_2_ capture in gas–liquid membrane contactors.

## 4. Conclusions

F-SiC nanoparticles were successfully incorporated into polysulfone hollow fiber membranes to improve their performance for CO_2_ absorption in gas–liquid membrane contactors. Increasing the F-SiC loading enhanced membrane hydrophobicity, increasing the water contact angle from 77.5° for the pristine membrane to 107.0° for the S6 membrane, while the critical entry pressure of water increased from 4.0 to 10.0 bar, indicating substantially improved resistance to pore wetting. Membrane densification reduced the average pore size from 161.67 to 77.96 nm and increased the mechanical collapse pressure from 4.0 × 10^5^ to 11.0 × 10^5^ Pa. CO_2_ absorption experiments demonstrated that increasing the absorbent flow rate enhanced CO_2_ flux for both physical and reactive absorption systems by reducing the liquid-side mass-transfer resistance. Among all fabricated membranes, the S4 membrane (4 wt.% fluorinated SiC) exhibited the optimum overall performance. Using distilled water, the maximum CO_2_ absorption flux reached 7.6 × 10^−4^ mol m^−2^ s^−1^, whereas a significantly higher value of 1.59 × 10^−3^ mol m^−2^ s^−1^ was obtained with 1 M NaOH, representing approximately a 2.3-fold improvement over the pristine membrane under identical operating conditions. Wilson plot analysis further confirmed that the S4 membrane possessed the lowest total operational mass-transfer resistance (dominated by a substantially reduced liquid-film resistance term), demonstrating that moderate F-SiC incorporation provides the best compromise between membrane hydrophobicity, wetting resistance, pore structure, and gas permeability. Long-term stability tests further verified the superior anti-wetting performance of the S4 membrane. The onset of CO_2_ flux decline was delayed from 35 h for the pristine membrane to 125 h for the S4 membrane, while the flux reduction after 145 h of continuous operation was limited to only 12.25%, compared with 44.44% for the pristine membrane. These results confirm that F-SiC nanoparticles effectively suppress membrane pore wetting and maintain stable gas–liquid mass transfer during prolonged operation. Although higher nanoparticle loading further increased hydrophobicity, excessive membrane densification in the S6 membrane increased diffusion resistance and slightly reduced CO_2_ absorption performance. However, S6 was not included in the long-term stability testing, so whether its higher contact angle (107.0° vs. 91.6°) and CEPw (10.0 vs. 9.5 bar) would translate into superior long-term wetting resistance relative to S4 remains an open question and is recommended as a priority for future work. Within the conditions evaluated in this study, the optimized S4 membrane combines high CO_2_ absorption flux, strong wetting resistance, low overall mass-transfer resistance, and demonstrated long-term operational stability relative to the pristine membrane, making fluorinated SiC-modified PSf hollow fiber membranes prom-ising candidates for durable and efficient CO_2_ capture in gas–liquid membrane contactor applications.

While the present study establishes the structure–property relationships and the short- and long-term CO_2_ absorption performance of fluorinated SiC/PSf hollow fiber membranes under controlled laboratory conditions, several directions remain important for advancing this membrane system toward practical applications. First, evaluation under realistic multi-component flue gas conditions (including N_2_, O_2_, SO_2_, and NO_x_) is needed to assess mixed-gas selectivity and competitive absorption behavior, and is currently being planned as a subsequent study. Second, extended long-term stability testing using 1 M NaOH and other reactive absorbents would establish the combined resistance of the membrane to chemical exposure and pore wetting under conditions more representative of industrial CO_2_ capture, complementing the distilled-water stability results reported here. Third, scale-up from the small laboratory contactor module used in this work to a larger multi-fiber module operated under continuous solvent circulation and regeneration would be required to establish performance and durability at a process-relevant scale and liquid-to-gas ratio. Finally, the F-SiC modification strategy demonstrated here may be extendable to other emerging membrane contactor applications, including direct air capture, biogas upgrading, and natural gas sweetening, where membrane wetting resistance and mechanical durability are similarly critical; incorporating advanced nanofiller chemistries such as MXenes or covalent organic frameworks alongside the fluorinated-filler approach used in this work represents a promising avenue for further improving gas transport and anti-wetting performance in future membrane designs.

## Figures and Tables

**Figure 1:**
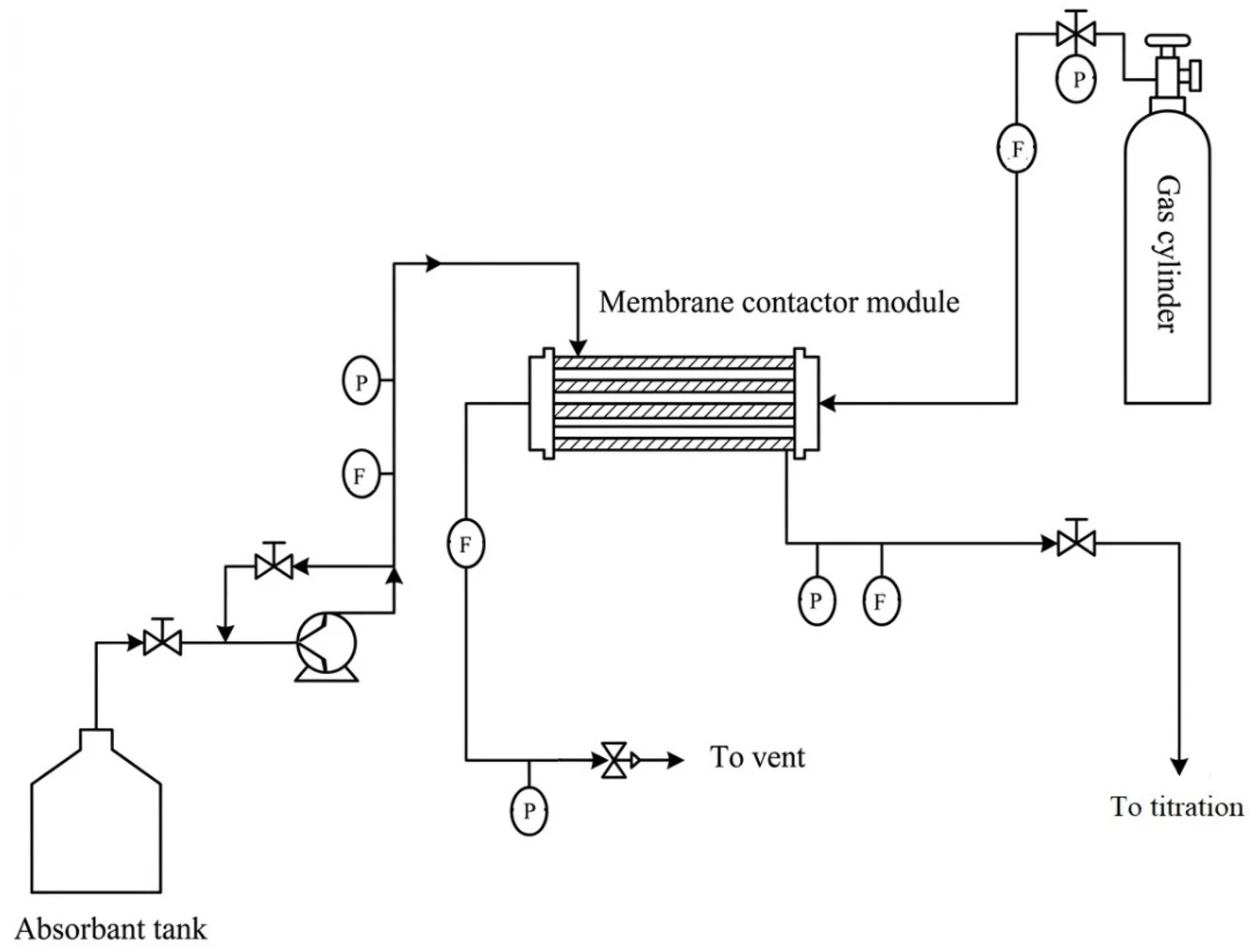
Schematic diagram of the experimental membrane contactor setup used for CO_2_ absorption.

**Figure 2:**
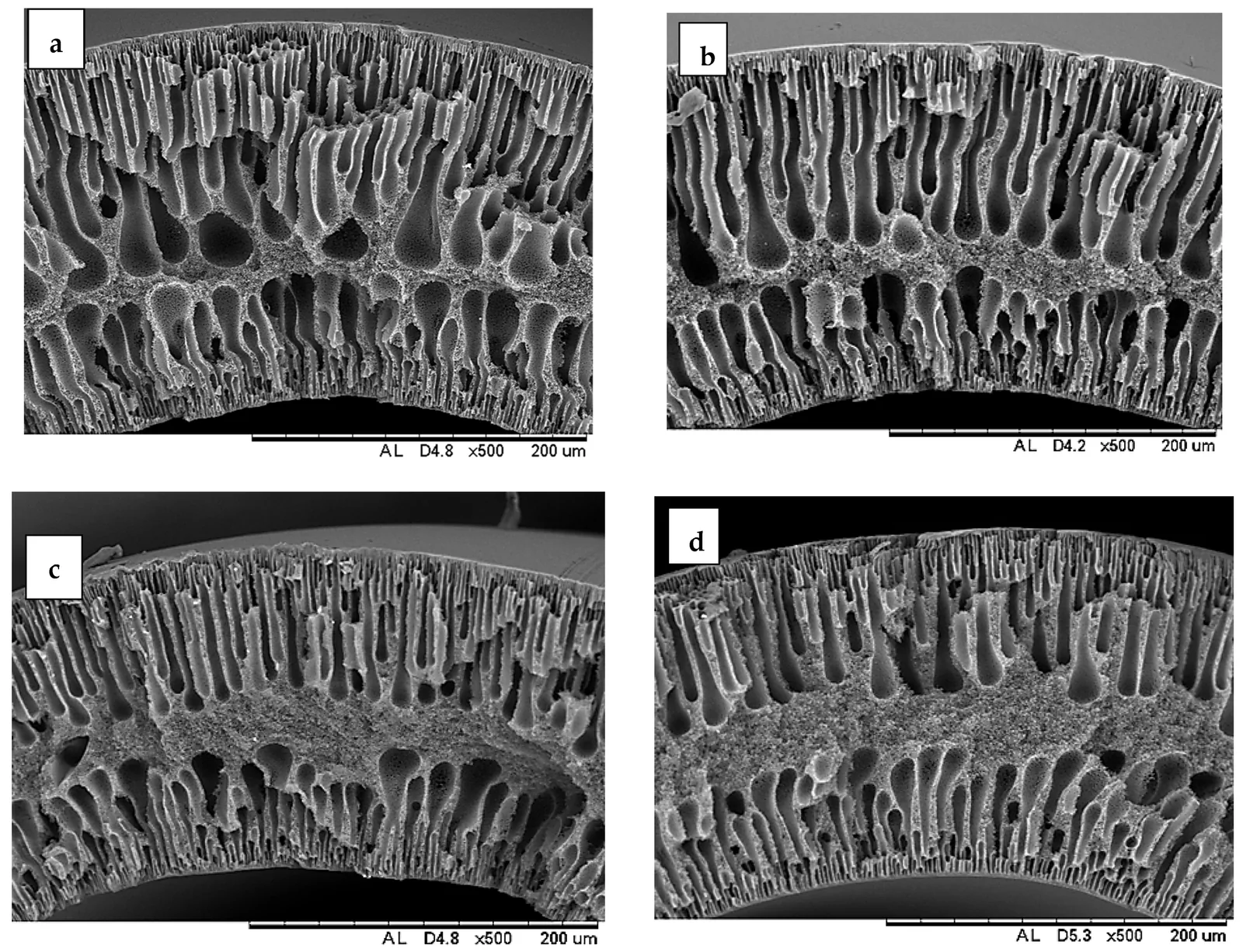
Cross-Sectional morphology of the PSf/F-SiC hollow fiber membranes with different nanoparticle loadings: (**a**) 0 wt.%, (**b**) 2 wt.%, (**c**) 4 wt.%, and (**d**) 6 wt.%.

**Figure 3:**
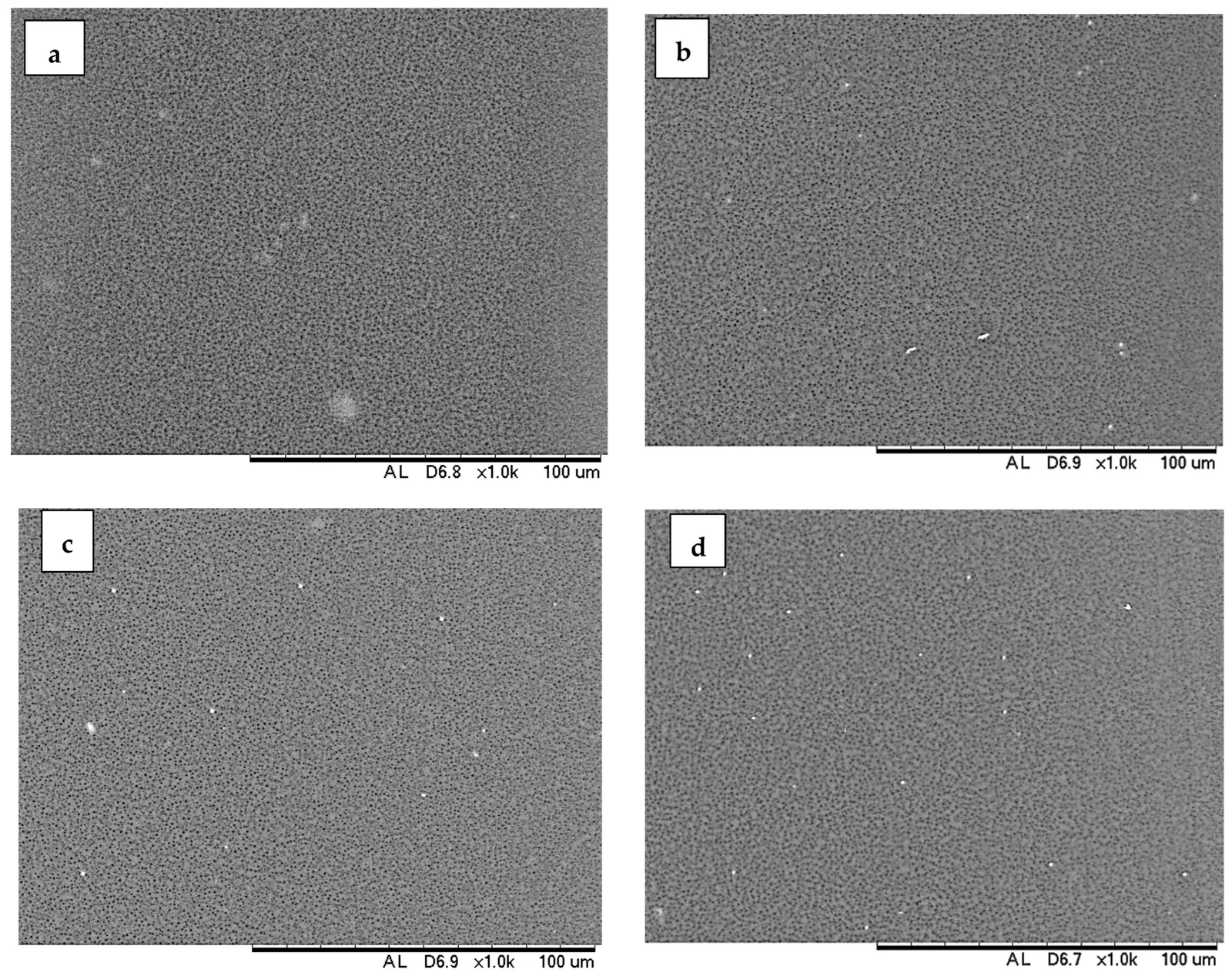
SEM micrographs of the inner lumen surfaces of the PSf/F-SiC hollow fiber membranes with different nanoparticle loadings: (**a**) 0 wt.%, (**b**) 2 wt.%, (**c**) 4 wt.%, and (**d**) 6 wt.%.

**Figure 4:**
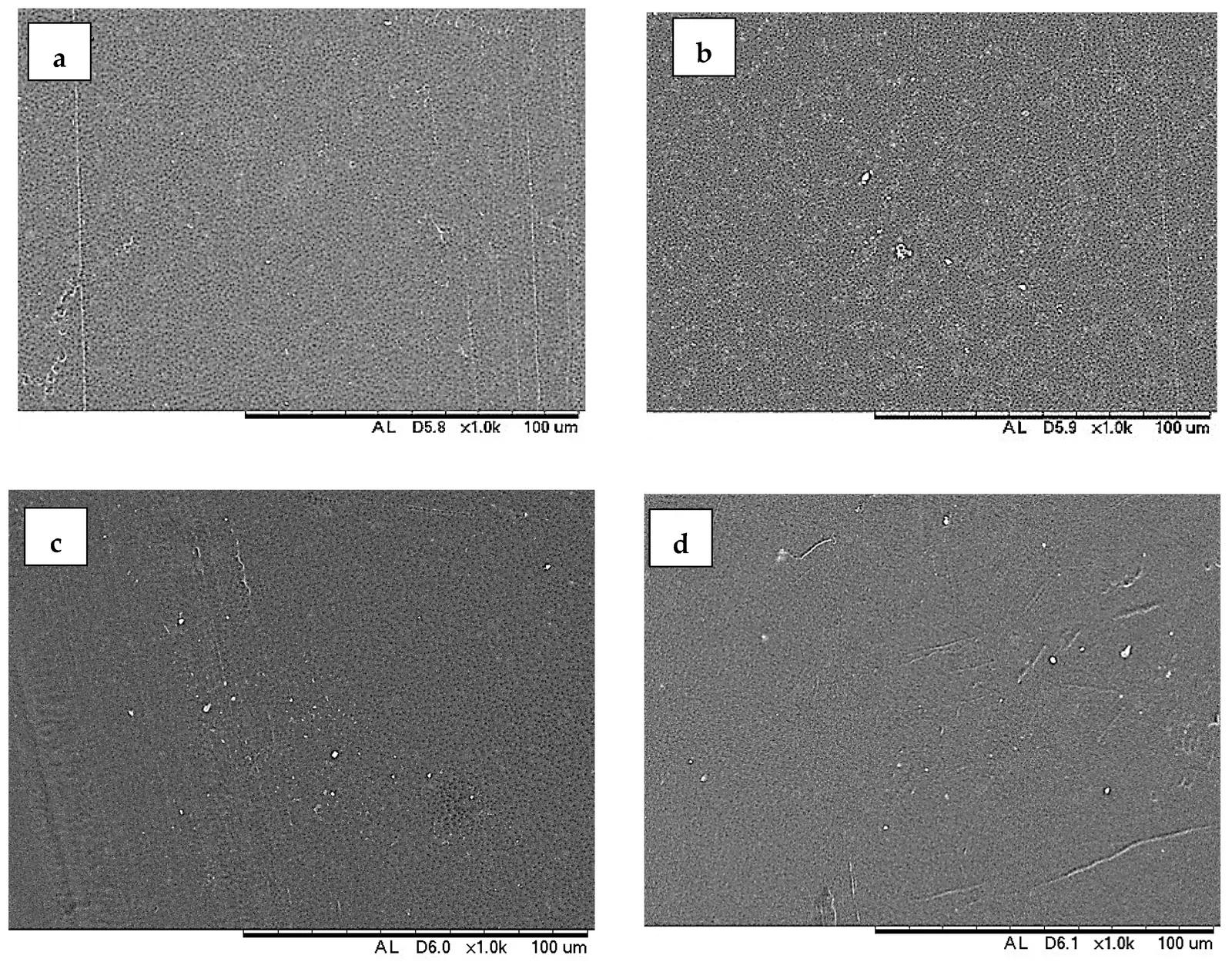
SEM micrographs of the outer surfaces of the PSf/F-SiC hollow fiber membranes fabricated via wet spinning with different nanoparticle loadings: (**a**) 0 wt.%, (**b**) 2 wt.%, (**c**) 4 wt.%, and (**d**) 6 wt.%.

**Figure 5:**
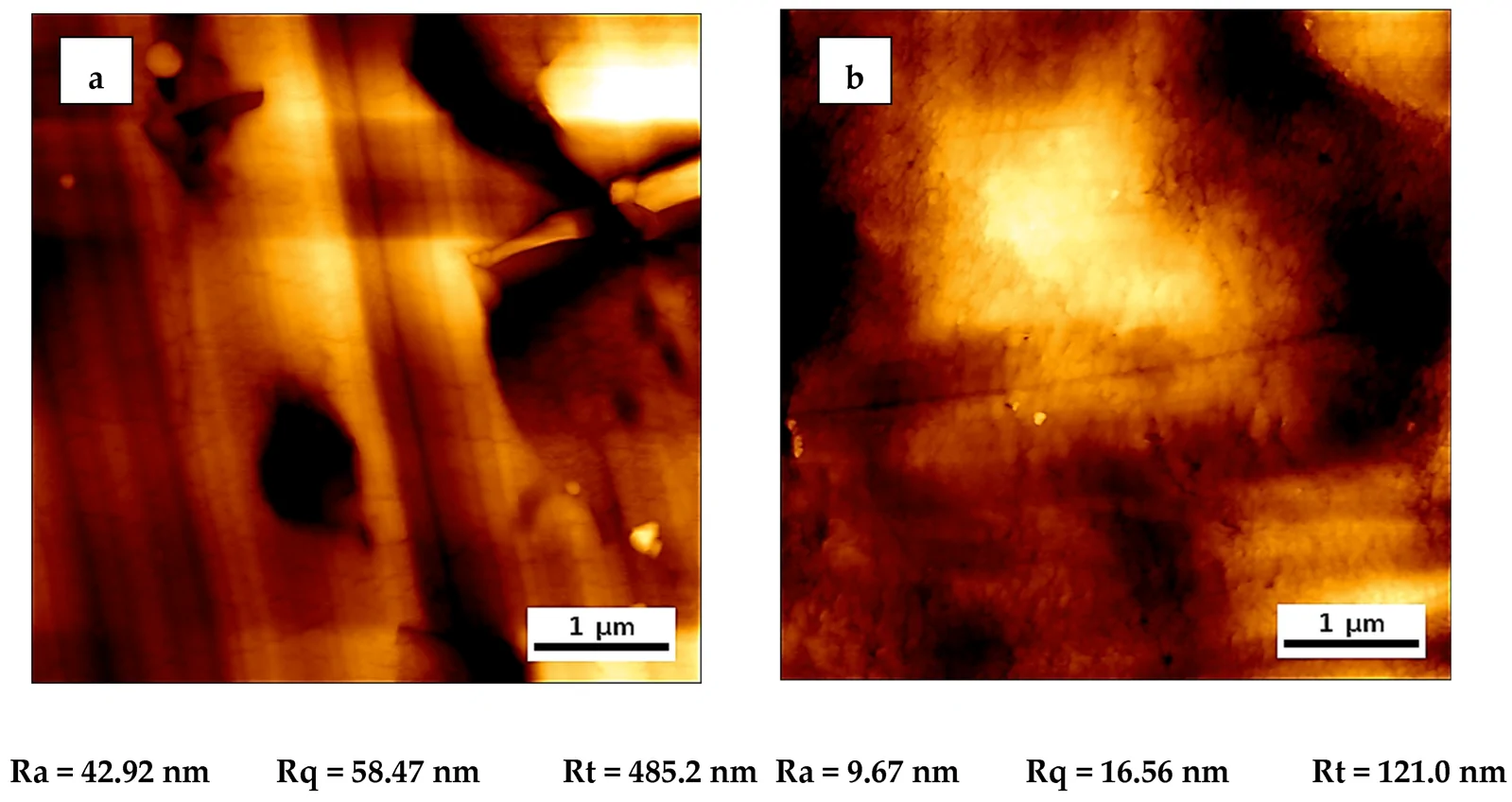

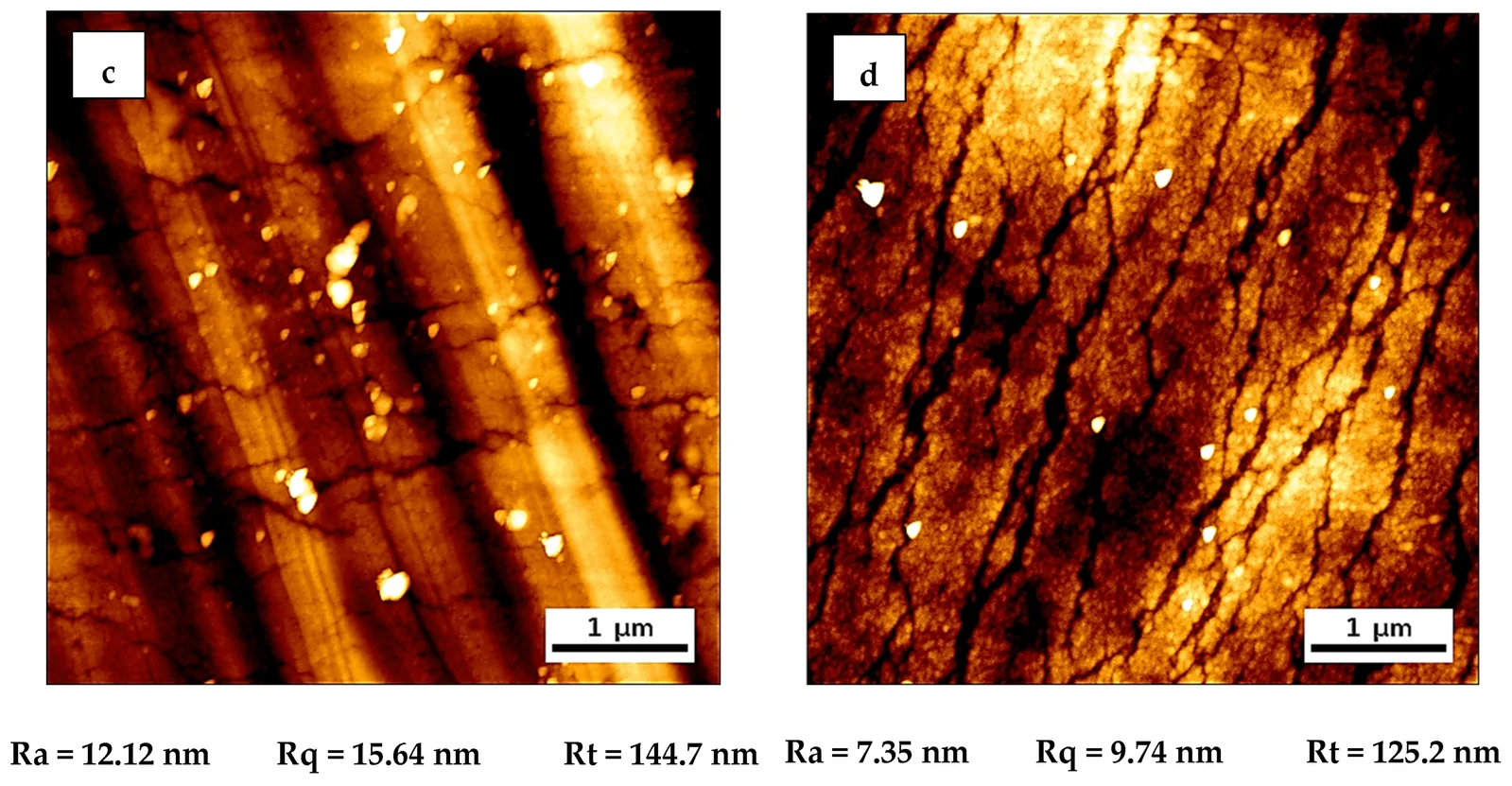
The 2D AFM topography scans of the outer surfaces of the fabricated PSf/F-SiC hollow fiber membranes: (**a**) 0 wt.%, (**b**) 2 wt.%, (**c**) 4 wt.%, and (**d**) 6 wt.% nanoparticle loading (scan area: 5 µm × 5 µm).

**Figure 6:**
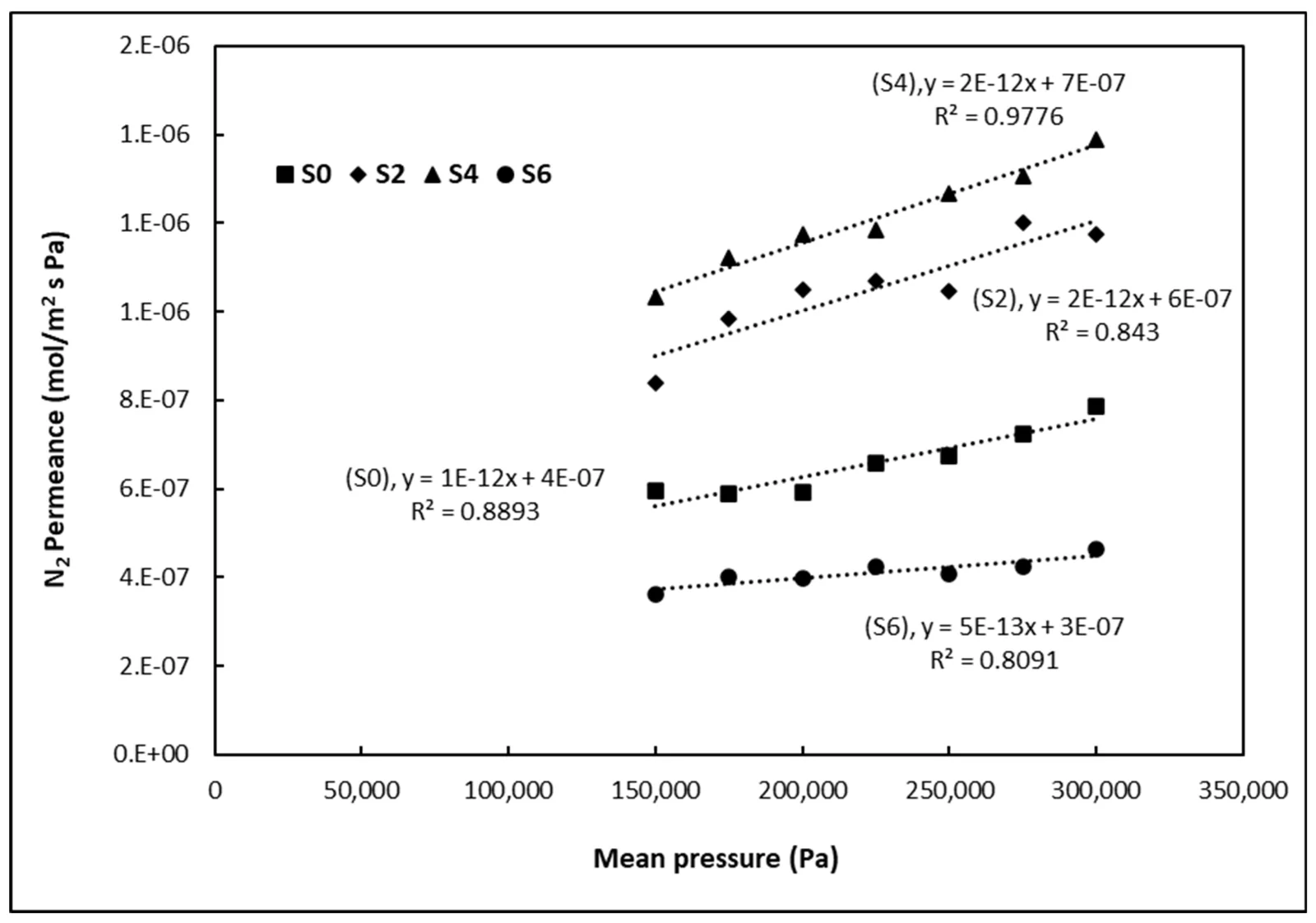
N_2_ permeance of the fabricated hollow fiber membranes at varying mean transmembrane pressures.

**Figure 7:**
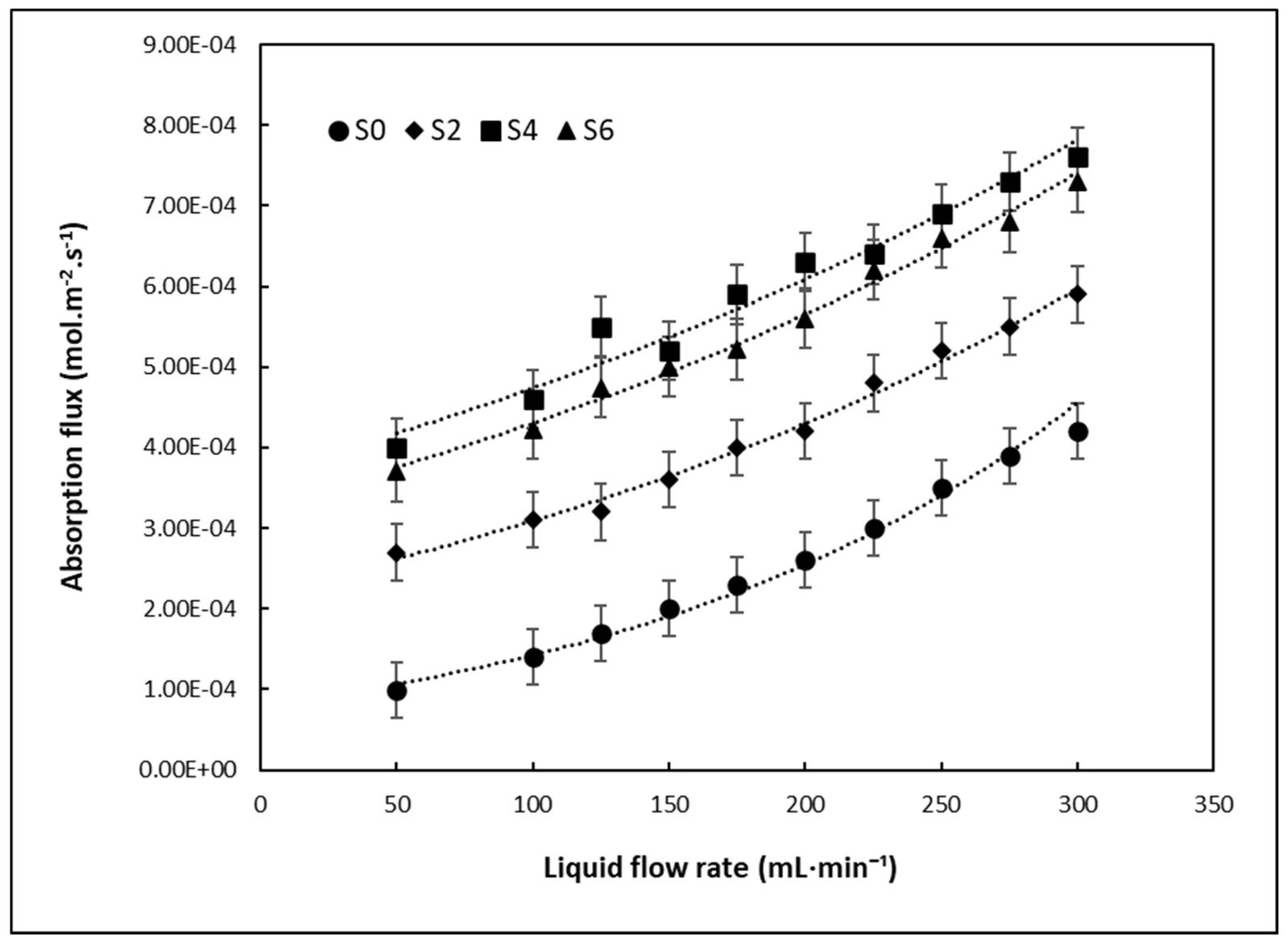
Effect of distilled water flow rate on the CO_2_ absorption flux of gas–liquid membrane contactors.

**Figure 8:**
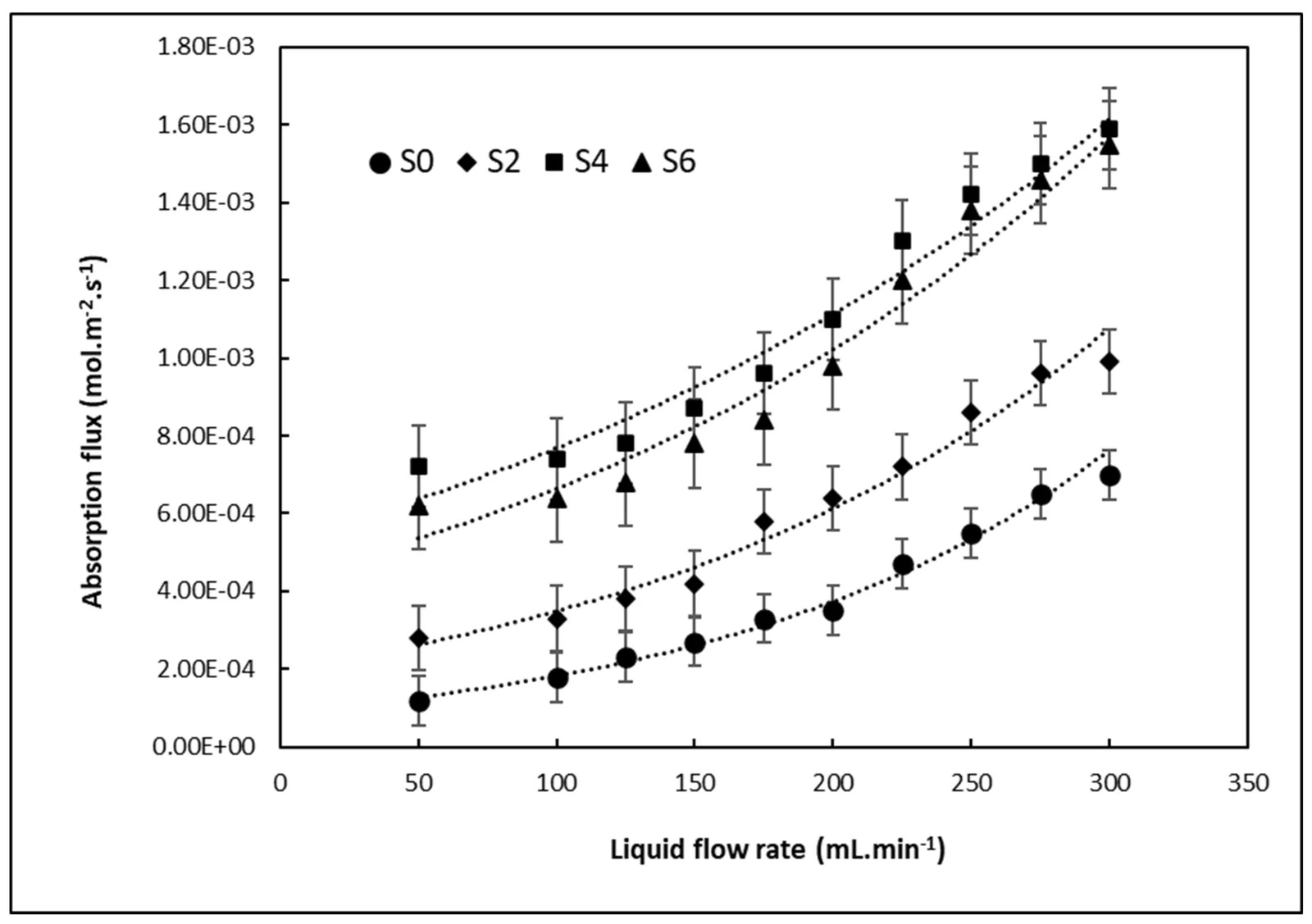
Effect of NaOH absorbent flow rate (1 M) on CO_2_ absorption performance in gas–liquid membrane contactors.

**Figure 9:**
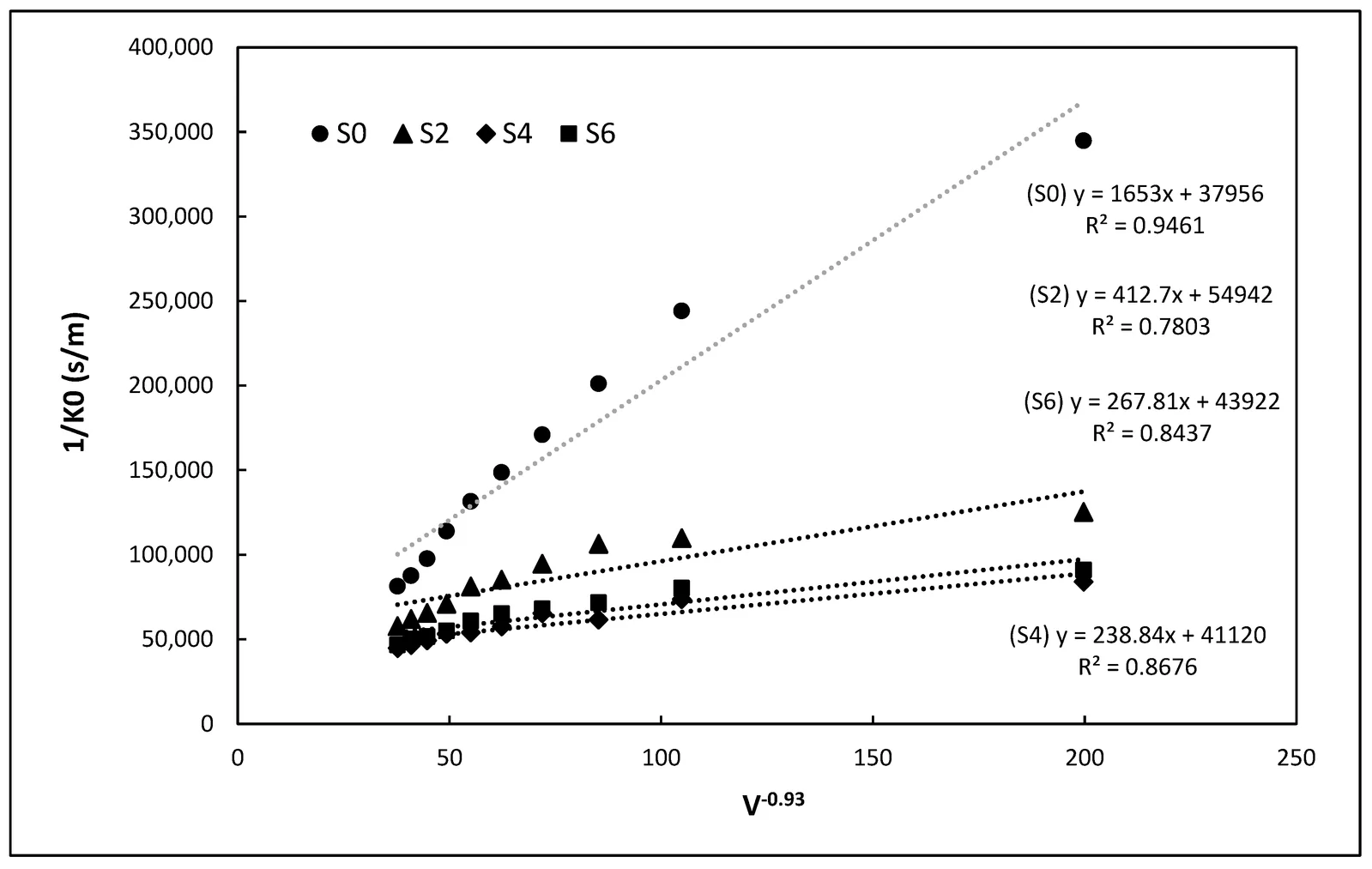
Wilson plots illustrating CO_2_ absorption through PSf hollow fiber membranes. (pure CO_2_–distilled water system).

**Figure 10:**
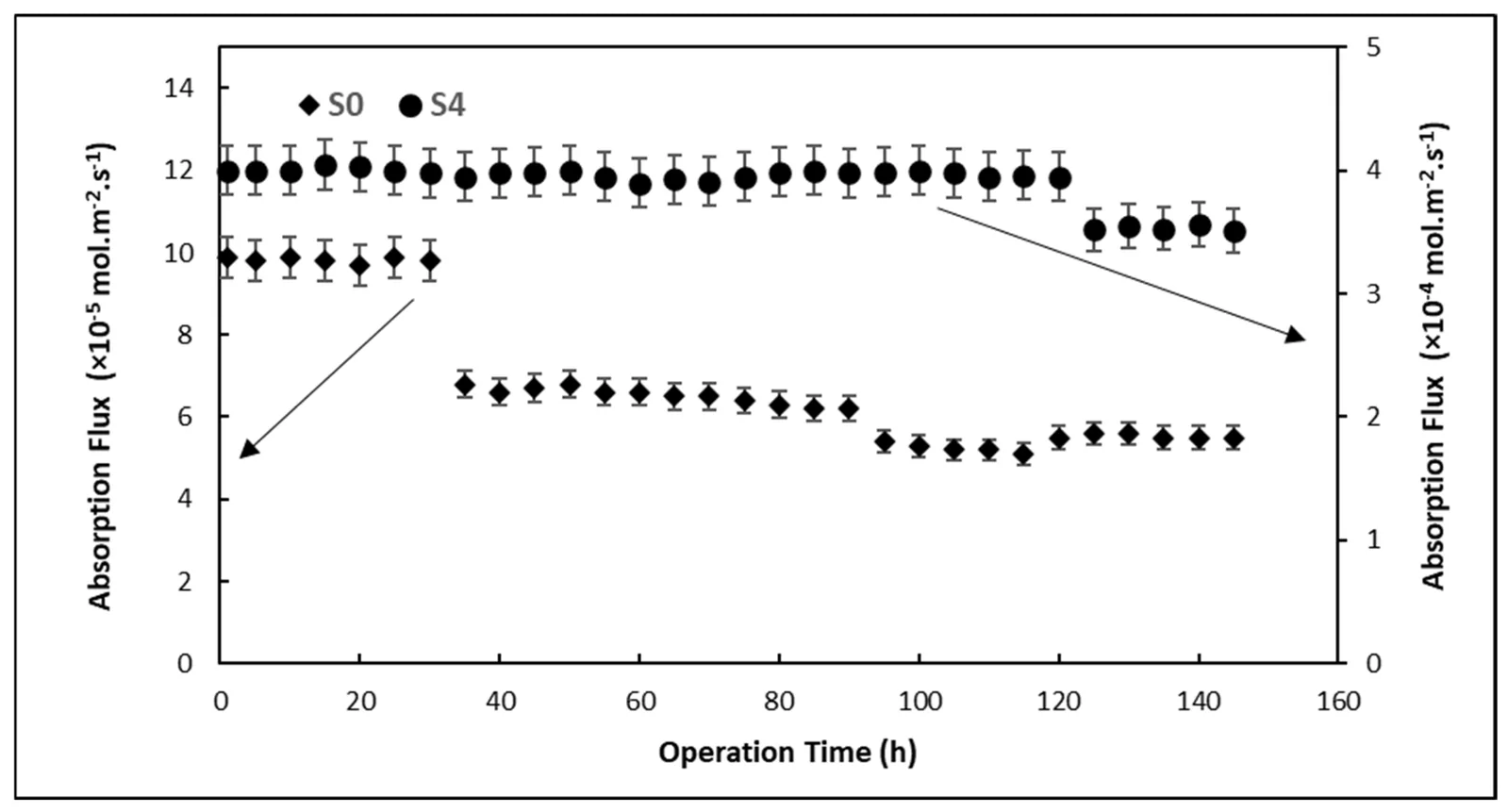
Long-term CO_2_ absorption behavior of S0 and S4 hollow fiber membrane contactors using distilled water at liquid and gas flow rates of 50 and 100 mL min^−1^, respectively. Error bars represent standard deviation (N = 3).

**Table 1:** Formulation and viscosity of polymer solutions.

Sample	PSf (wt.%)	F-SiC (wt.%)	NMP (wt.%)	Viscosity (cP)
S0	18	0	82	1887 ± 35
S2	18	2	80	2273 ± 35
S4	18	4	78	2887 ± 65
S6	18	6	76	3597 ± 91

**Table 2:** Spinning conditions employed for the preparation of hollow fiber membranes.

Dope extrusion rate (mL/min)	4.0
Bore fluid composition, NMP/water (wt.%)	90/10
Bore fluid rate (mL/min)	2.0
External coagulant	Tap water
Air gap distance (cm)	0.0
Spinneret o.d/i.d (mm)	1.0/0.50
Coagulation temperature (°C)	25

**Table 3:** Specifications of the gas–liquid membrane contactor setup [17].

Module i.d. (mm)	18
Module length (mm)	300
Fiber o.d. (mm)	0.85–0.95
Fiber i.d (mm)	0.40–0.45
Effective fiber length (mm)	200
Number of fibers	10

**Table 4:** Elemental composition (wt.%) of the pristine PSf and F-SiC modified hollow fiber membranes obtained via EDX analysis.

Element	wt.%			
	S0	S2	S4	S6
C	77.20	75.80	74.30	72.80
O	15.30	15.00	14.60	14.10
S	7.50	7.30	7.10	6.80
Si	0.00	1.10	2.30	3.60
F	0.00	0.80	1.70	2.70
Total	100.00	100.00	100.00	100.00

**Table 5:** Physical, structural, mechanical, and wetting properties of pristine PSf and F-SiC modified mixed-matrix hollow fiber membranes.

Membrane	Water Contact Angle (°)	CEPw (Bar)	Average Pore Size (nm)	Effective Surface Porosity, ε/Lp (m^−1^)	Collapsing Pressure (×10^5^ Pa)	Overall Porosity (%)
S0	77.50 ± 2	4.00 ± 0.50	161.67	17.66	4.00	78.5 ± 2
S2	86 ± 1	6.50 ± 0.50	155.22	29.91	8.50	74.1 ± 2
S4	91.60 ± 2	9.50 ± 0.50	138.33	40.54	10.50	70.6 ± 2
S6	107.0 ± 3	10.0 ± 0.50	77.96	29.75	11.00	69.3 ± 2

**Table 6:** The Wilson-plot regression parameters and resolved mass-transfer resistance components for F-SiC modified PSf hollow fiber membranes.

Membrane	Slope (Liquid-Film Coefficient, s·m^−1^·(m/s)^0.93^)	Intercept—Membrane Resistance, 1/k_m (s/m)	R^2^	Liquid-Film Resistance at 50 mL min^−1^ (s/m)	Total Resistance 1/K_0_ at 50 mL min^−1^ (s/m)	Membrane Share of Total (%)
S0	1653.0	37,956	0.946	330,158	368,114	10.3%
S2	412.7	54,942	0.780	82,430	137,372	40.0%
S4	238.8	41,120	0.868	47,704	88,824	46.3%
S6	267.8	43,922	0.844	53,490	97,412	45.1%

**Table 7:** Comparison of the performance of the present fluorinated SiC/PSf hollow fiber membrane with previously reported membrane contactors for gas absorption applications.

Membrane/Modification	Target Gas	Absorbent	Water Contact Angle (°)	CEPw/LEPw (Bar)	Maximum Gas Absorption Flux (mol m^−2^ s^−1^)	Key Findings	Advantages Compared with Current Study	Ref.
PVDF/MMT MMM (1 wt.% MMT)	CO_2_	Distilled water	90	9.5	1.0 × 10^−3^	Improved hydrophobicity and wetting resistance; 82% higher flux than pristine membrane.	Comparable wetting resistance, but lower hydrophobicity and lower CO_2_ flux than NaOH-assisted absorption in this study.	[31]
PEI/MMT MMM (1 wt.% MMT)	CO_2_	Distilled water	86	5.5	2.35 × 10^−3^	Enhanced porosity, LEPw and mechanical stability with nano-clay incorporation.	Higher flux with water absorbent, but significantly lower hydrophobicity and wetting resistance than the F-SiC membrane.	[32]
PVDF/Cu@4A MMM	SO_2_	NaOH aqueous solution	—	—	9.1 × 10^−4^	Hierarchical Cu@4A promoted facilitated SO_2_ transport and reduced membrane resistance.	Different gas system; present work provides higher wetting resistance and investigates both physical and reactive CO_2_ absorption.	[16]
Superhydrophobic PES/SiO_2_ coated membrane	SO_2_	Distilled water	159	1.389	1.24 × 10^−3^	Lotus-leaf morphology; excellent superhydrophobicity and stable SO_2_ removal.	Much higher contact angle, but LEPw is substantially lower. Present membrane achieves far superior wetting resistance (10 bar) with high CO_2_ absorption performance.	[33]
PPO/SiO_2_ MMM	CO_2_	Monoethanolamine	142.9	—	5.8 × 10^−3^	Excellent CO_2_ flux and 20-day stability using flat-sheet membrane contactor.	Higher CO_2_ flux owing to different membrane geometry and operating conditions; however, current study demonstrates superior hollow-fiber wetting resistance and evaluates membrane mass-transfer resistance using Wilson analysis.	[34]
PVDF/MWCNT MMM	CO_2_	Al_2_O_3_ nanofluid absorbent	103	—	3.85 × 10^−3^	Enhanced pore structure and long-term stability using Al_2_O_3_ nanofluid absorbent.	Higher flux achieved using nanofluid absorbent and different operating conditions. Current study uses conventional absorbents while achieving higher CEPw (10 bar) and comprehensive membrane characterization.	[35]
ZnO–NH_2_ nanofluid absorbent	CO_2_	Water-based and MDEA-based nanofluid absorbents	—	—	Flux enhancement of 68.2% (water) and 12.7% (MDEA)	Functionalized nanofluid improved liquid-side mass transfer without membrane modification.	Focuses on absorbent optimization rather than membrane development. Present study enhances membrane properties and directly reduces membrane mass-transfer resistance.	[36]
Fluorinated SiC/PSf MMM (S4)	CO_2_	Distilled water and 1.0 M aqueous NaOH	91.6	9.5	7.6 × 10^−4^ (water) 1.59 × 10^−3^ (1 M NaOH)	F-SiC simultaneously enhanced hydrophobicity, wetting resistance, mechanical strength, and reduced membrane mass-transfer resistance. Wilson plot analysis identified an optimum filler loading (4 wt.%) balancing permeability and wetting resistance.	High CEPw (9.5–10 bar), improved mechanical stability, reduced membrane resistance, dual evaluation using physical and chemical absorbents, and systematic optimization of F-SiC loading in hollow-fiber membranes.	This work

## Data Availability

The data supporting the findings of this study are available from the corresponding author upon reasonable request.
